# Opposing Regulation of the EGF Receptor: A Molecular Switch Controlling Cytomegalovirus Latency and Replication

**DOI:** 10.1371/journal.ppat.1005655

**Published:** 2016-05-24

**Authors:** Jason Buehler, Sebastian Zeltzer, Justin Reitsma, Alex Petrucelli, Mahadevaiah Umashankar, Mike Rak, Patricia Zagallo, Joyce Schroeder, Scott Terhune, Felicia Goodrum

**Affiliations:** 1 BIO5 Institute, University of Arizona, Tucson, Arizona, United States of America; 2 Department of Cellular and Molecular Medicine, University of Arizona, Tucson, Arizona, United States of America; 3 Department of Microbiology and Molecular Genetics, Medical College of Wisconsin, Milwaukee, Wisconsin, United States of America; 4 Department of Immunobiology, University of Arizona, Tucson, Arizona, United States of America; 5 Department of Molecular and Cellular Biology, University of Arizona, Tucson, Arizona, United States of America; 6 University of Arizona Cancer Center, University of Arizona, Tucson, Arizona, United States of America; Louisiana State University Health Sciences Center, UNITED STATES

## Abstract

Herpesviruses persist indefinitely in their host through complex and poorly defined interactions that mediate latent, chronic or productive states of infection. Human cytomegalovirus (CMV or HCMV), a ubiquitous β-herpesvirus, coordinates the expression of two viral genes, *UL135* and *UL138*, which have opposing roles in regulating viral replication. *UL135* promotes reactivation from latency and virus replication, in part, by overcoming replication-suppressive effects of *UL138*. The mechanism by which *UL135* and *UL138* oppose one another is not known. We identified viral and host proteins interacting with *UL138* protein (pUL138) to begin to define the mechanisms by which pUL135 and pUL138 function. We show that pUL135 and pUL138 regulate the viral cycle by targeting that same receptor tyrosine kinase (RTK) epidermal growth factor receptor (EGFR). EGFR is a major homeostatic regulator involved in cellular proliferation, differentiation, and survival, making it an ideal target for viral manipulation during infection. pUL135 promotes internalization and turnover of EGFR from the cell surface, whereas pUL138 preserves surface expression and activation of EGFR. We show that activated EGFR is sequestered within the infection-induced, juxtanuclear viral assembly compartment and is unresponsive to stress. Intriguingly, these findings suggest that CMV insulates active EGFR in the cell and that pUL135 and pUL138 function to fine-tune EGFR levels at the cell surface to allow the infected cell to respond to extracellular cues. Consistent with the role of pUL135 in promoting replication, inhibition of EGFR or the downstream phosphoinositide 3-kinase (PI3K) favors reactivation from latency and replication. We propose a model whereby pUL135 and pUL138 together with EGFR comprise a molecular switch that regulates states of latency and replication in HCMV infection by regulating EGFR trafficking to fine tune EGFR signaling.

## Author Summary

Cytomegalovirus, a herpesvirus, persists in its host through complex interactions that mediate latent, chronic or productive states of infection. Defining the mechanistic basis viral persistence is important for defining the costs and possible benefits of viral persistence and to mitigate pathologies associated with reactivation. We have identified two genes, *UL135* and *UL138*, with opposing roles in regulating states of latency and replication. *UL135* promotes replication and reactivation from latency, in part, by overcoming suppressive effects of *UL138*. Intriguingly, pUL135 and pUL138 regulate the viral cycle by targeting the same receptor tyrosine kinase, epidermal growth factor receptor (EGFR). EGFR is a major homeostatic regulator controlling cellular proliferation, differentiation, and survival, making it an ideal target for viruses to manipulate during infection. We show that CMV insulates and regulates EGFR levels and activity by modulating its trafficking. This work defines a molecular switch that regulates latent and replicative states of infection through the modulation of host trafficking and signaling pathways. The regulation of EGFR at the cell surface provides a novel means by which the virus may sense and respond to changes in the host environment to enter into or exit the latent state.

## Introduction

Human cytomegalovirus (CMV), a β-herpesvirus ubiquitous in the world’s population, has adapted many trade-offs for its persistence. CMV replicates to low titers and causes minimal cytopathology, such that the primary infection is typically unapparent. CMV, like all herpesviruses, persists in the host through the establishment of latent state and chronic states [1]. During latency, CMV genomes are maintained in the infected cell with little to no viral gene expression and no virus replication. Given the commitment of T-cell immunity to CMV infection [2], CMV likely reactivates subclinically with high frequency. However, in an immune incompetent host, including solid organ or stem cell transplant recipients, CMV reactivation remains a major cause of morbidity and mortality [3]. There is no CMV vaccine, and current antivirals fail to target the latent virus. Understanding the mechanistic basis of latency is critical to developing strategies to target latent virus.

The UL*b*’ region of the CMV genome is conserved between human, chimpanzee and rhesus macaque CMV strains, but is completely lacking from rat, mouse and guinea pig strains, suggesting that this region represents an adaptation of the virus to the primate host [4]. The UL*b*’ region is lost upon serial passage of the virus in fibroblasts [5], resulting in viruses with higher replicative capacity but more restricted tropism. It is suspected that the estimated 20 open reading frames encoded by the UL*b*’ region are required for infection and persistence in the host. The *UL133-UL138* locus, termed *UL133/8*, is encoded within the UL*b*’ region and encodes four genes: *UL133*, *UL135*, *UL136* and *UL138*. These genes have important functions for replication in vascular endothelial cells [6, 7] and differentially regulate latency and reactivation in CD34^+^ hematopoietic progenitor cells (HPCs) [4, 8–10], a site of CMV latency. Antagonism between *UL135* and *UL138* highlights the complex interplay between proteins encoded by the *UL133/8* locus in regulating levels of replication. *UL138* suppresses virus replication and promotes latency in CD34^+^ HPCs [4, 8, 11]. By contrast, *UL135* promotes *de novo* replication from transfected viral genomes when *UL138* is expressed and is required for reactivation from latency in CD34^+^ HPCs. Thus, *UL135* functions, in part, by overcoming the suppressive effects of *UL138* [10]. These studies suggest the existence of a genetic switch regulating states of infection; however, the mechanism by which *UL135* and *UL138* regulate infection states is unknown.

In this study, we demonstrate that *UL138* and *UL135* proteins (pUL138 and pUL135) antagonize one another by targeting EGFR. EGFR is a powerful host target as it regulates cellular proliferation, differentiation, angiogenesis and survival [12]. While pUL138 potentiates EGFR signaling by enhancing cell surface levels, pUL135 diminishes EGFR signaling by promoting its turnover. The opposing dual targeting of EGFR by pUL135 and pUL138 suggests that modulation of receptor tyrosine kinase (RTK) trafficking and signaling underlies, at least in part, the transition of the virus into and out of latency. Indeed inhibition of EGFR or downstream PI3K favors viral replication and stimulates reactivation of *UL135*-mutant viruses in CD34^+^ HPCs. These studies define a genetic switch regulating viral replication and latency in the host.

## Results

### pUL135 and pUL138 interact with EGFR

**Fig 1:**
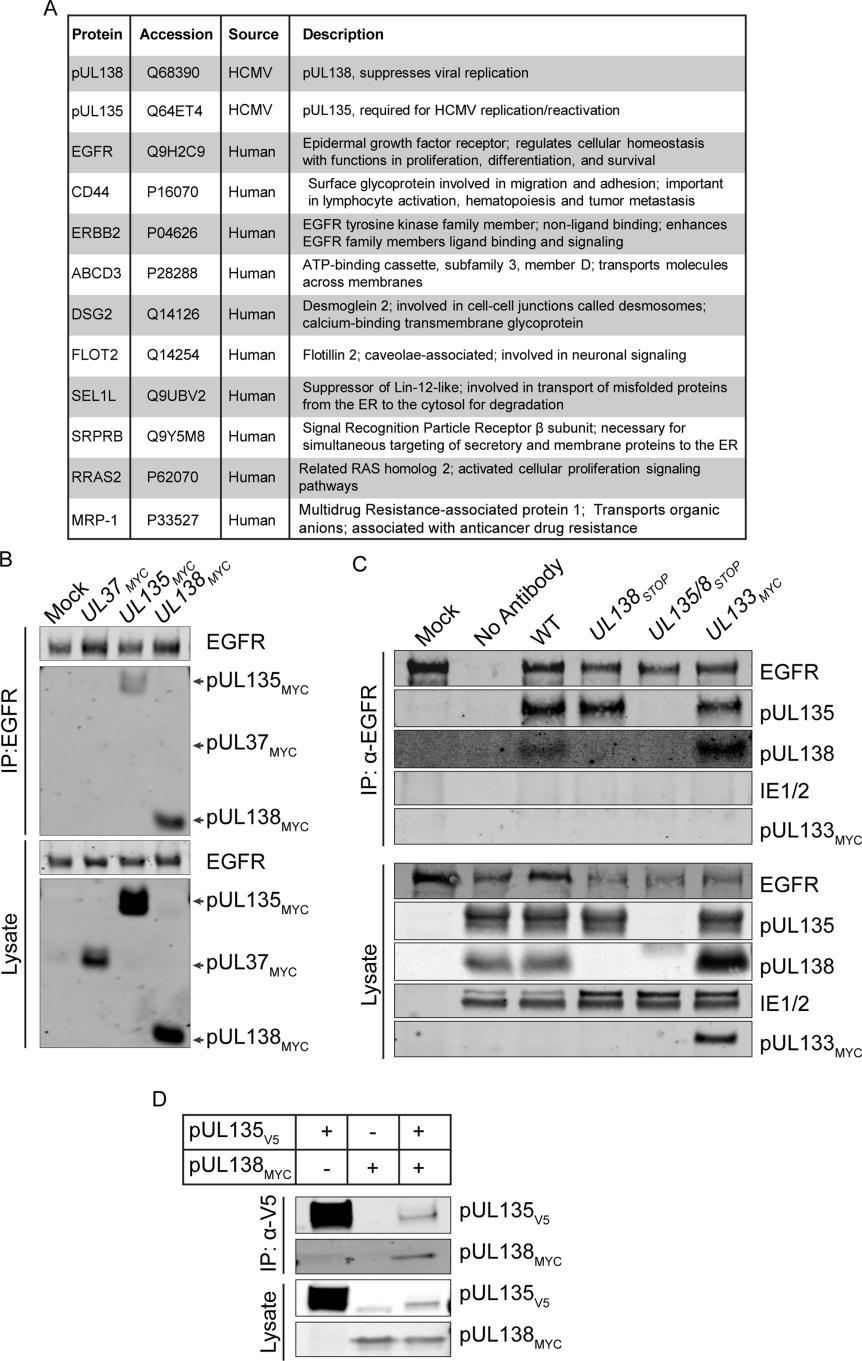
pUL138 and pUL135 interact with EGFR. (A) pUL138 fused with a C-terminal 3XFlag epitope tag was immunoprecipitated with a Flag-specific antibody from lysates derived from fibroblasts infected with TB40/E-*UL138*_*3XFLAG*_ at 48 hpi. Following tryptic digest, peptides were identified by LC-MS/MS. Candidates were subtracted from a parallel Flag antibody pull-down from infected cell lysates without a Flag-tagged protein. 128 total candidates remained after subtraction. High priority candidates belonging to a network of interactions were identified by STRING and NCBI analysis. UL138 interacted with EGFR with a number of proteins associated with EGFR signaling. (B) Interaction between EGFR and either pUL135 or pUL138 was confirmed by the reciprocal co-immunoprecipitation. Fibroblasts were transduced with lentiviruses expressing *UL135*_*MYC*_, *UL138*_*MYC*_ or *UL37*_*MYC*_ (control). EGFR was precipitated using ms α-EGFR and interactions were detected by blotting with chk α-myc or rb α-EGFR. (C) EGFR was immunoprecipitated from lysates derived from fibroblasts mock-infected or infected with 1 MOI of WT, *UL138*_STOP_, *UL135/8*_STOP_, or *UL133*_MYC_ (control). Interactions were detected by blotting for rb α-pUL135, rb α-pUL138, and chk α-myc. D. pUL135_V5_ was immunoprecipitated using ms α-V5 from lystates derived from HEK cells overexpressing pUL135_V5_, pUL138_MYC_, or both. Interactions were detected by immunoblotting with chk α-myc and chk α-V5. For B-D, total lysates are shown.

We identified host interacting proteins by mass spectrometry following the immunoprecipitation of flag epitope-tagged pUL138 (IP-MS/MS) in the context of infection to begin to understand the mechanisms by which *UL138* suppresses viral replication. Top-ranking co-precipitating proteins based on peptide count and coverage are shown in Fig 1A. IP-MS/MS peptides and data are provided for these candidates in S1 Table. EGFR was a particularly interesting candidate because it sits at the center of a network of related pUL138-host interactions as determined by STRING and NCBI analysis, which are listed in Fig 1A. Indeed, this was the only large network that emerged from the 128 interactions identified. Work by others has demonstrated interactions between pUL138 and two other receptors, TNFR [13, 14] and MRP-1 [15]. Our study confirmed the interaction with MRP-1 (Fig 1A).

We confirmed the interaction between pUL138 and EGFR with a reciprocal pull-down, immunoprecipitating EGFR in cells transiently expressing Myc- tagged fusion proteins, pUL135_MYC_, pUL138_MYC_, and pUL37_MYC_. Consistent with the IP-MS/MS results, pUL138_MYC_ co-precipitated with EGFR (Fig 1B). Intriguingly, we also detected an interaction between pUL135_MYC_ and EGFR. These results suggest that both pUL138 and pUL135 interact with EGFR in the absence of other viral factors. The interaction between pUL138 or pUL135 with EGFR is not the result of the myc epitope tag because pUL37_MYC_ did not co-immunoprecipitate with EGFR. In the context of infection, we confirmed interaction between EGFR and pUL135 or pUL138, but not another myc-tagged protein from the UL133/8 locus, pUL133_MYC_ (Fig 1C). Viruses containing disruptions to prevent expression of *UL138* (*UL138*_STOP_) or *UL135* and UL138 (*UL135/8*_STOP_) serve as controls. These experiments indicate that both pUL135 and pUL138 interact with EGFR when expressed alone and in the context of infection. The interaction of both pUL135 and pUL138 with EGFR is intriguing given the antagonistic relationship between *UL135* and *UL138* in the context of infection [10].

Additionally, the IP-MS/MS screen indicated an interaction between pUL135 and pUL138, which confirms previous interactions studies (Fig 1A) [9]. To further investigate a requirement of EGFR for the interaction between pUL135 and pUL138, we overexpressed both pUL135_V5_ and pUL138_MYC_ in HEK-293 cells, which express little to no EGFR [16]. Immunoprecipitation of pUL135 (pUL135_V5_) using an antibody to the V5 tag co-precipitated pUL138_MYC_ (Fig 1D). This pull down is reciprocal to that of the IP-MS/MS experiment where pUL138_FLAG_ was pulled down (Fig 1A). The co-precipitation of pUL138 with pUL135 in HEK-293 cells suggests that the interaction between pUL135 and pUL138 does not require EGFR. Further work is required to define the domains of pUL135, pUL138, and EGFR required for interaction.

### pUL135 and pUL138 modulate EGFR surface levels during infection

**Fig 2:**
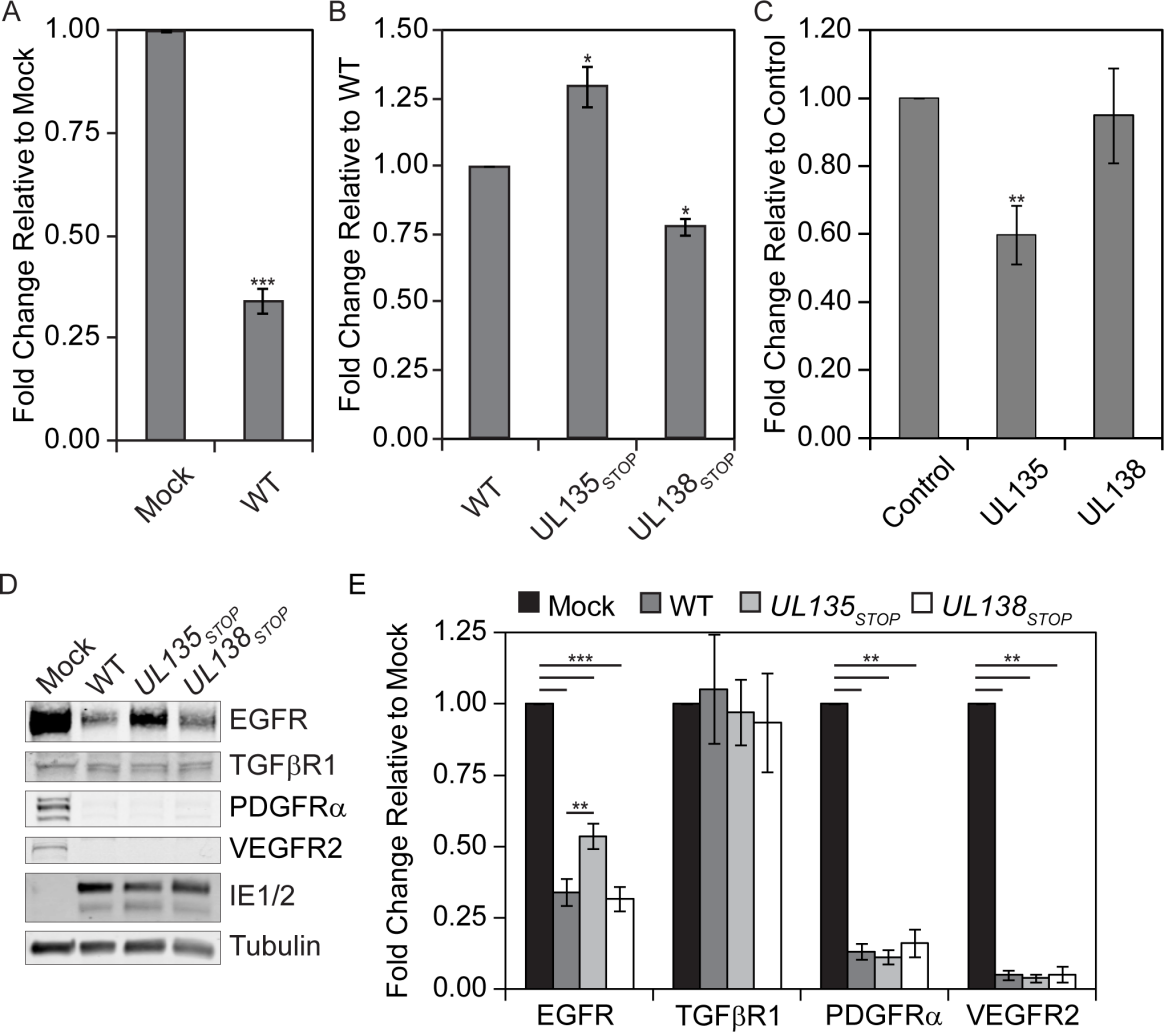
UL135 and UL138 alter EGFR surface levels. (A and B) Fibroblasts were left uninfected or infected with WT, *UL135*_STOP_, or *UL138*_*STOP*_ viruses at an MOI of 1. At 48 hpi, cells were stained with BV421 conjugated ms α-EGFR antibody and infected (GFP+) cells were analyzed by flow cytometry for differences in EGFR surface levels. (C) Fibroblasts were transduced with 1 MOI of control, *UL135*_*MYC*_, or *UL138*_*MYC*_ expressing lentivirus vector. Cells were stained with BV421 conjugated ms α-EGFR antibody and transduced (GFP+) cells were analyzed by flow cytometry for differences in EGFR surface levels. For A-C significance was calculated using a Student’s t-test. (D and E) Fibroblasts were infected with 1 MOI of WT, *UL135*_STOP_, and *UL138*_*STOP*_ virus and lysed at 48 hpi. Samples were separated by SDS-PAGE and blotted with rb α-EGFR, rb α-TGFβR1, ms α-IE1/2, rb α-PDGFRα, rb α-VEGFR2 and ms α-tubulin. In panel E, statistical significance was calculated by a one-way ANOVA with Tukey correction for each protein. EGFR and TGFβR1 values are calculated from six independent experiments, while PDGFRα and VEGFR2 were calculated from three experiments. Error bars represent standard error of the mean (SEM). Astrisk indicate p-values (* p-value ≤ 0.05; ** p-value ≤ 0.001; *** p-value ≤ 0.0001).

EGFR signaling may be modulated during viral infection in a number of ways, including phosphorylation, ubiquitination, and trafficking. pUL135 and pUL138 are membrane-associated proteins; pUL135 is localized at the Golgi, cell surface and cytoskeleton [4, 17], whereas pUL138 is at the Golgi [4, 8]. Because pUL138 has been shown to alter MRP-1 and TNFR at the cell surface [13–15], we wanted to determine if EGFR surface levels were altered during infection. EGFR was reduced by ~70% on the surface of WT-infected cells relative to mock-infected cells (p-value ≤ 0.001) (Fig 2A). This reduction is in part due to the reported transcriptional downregulation of EGFR during virus replication [18, 19]. Relative to WT infection, disruption of *UL135* increased EGFR surface levels by 29% (p-value ≤ 0.05), while disruption of *UL138* decreased EGFR surface levels by 22% (p-value ≤ 0.05) (Fig 2B). These data indicate an opposing role for pUL135 and pUL138 in modulating surface levels of EGFR.

We next asked if pUL135 and pUL138 affected cell surface levels of EGFR when expressed outside the context of viral infection (Fig 2C). *UL135* overexpression reduced EGFR surface levels compared to the control (Ratio of mean fluorescent intensities, MFI, 1.7±0.17), but *UL138* alone had no affect (Ratio of MFI, 1±0.28). This suggests that pUL135 alone stimulates reduction of EGFR levels at the cell surface, whereas pUL138 requires additional viral or infection-induced factors to stimulate EGFR expression at the cell surface. By contrast, *UL138* expression alone upregulates surface expression of TNFR1 [13, 14] and confirmed in S1 Fig, suggesting that UL138 requires other infection-specific factors for the regulation of EGFR, but not TNFR1.

The reduced surface expression of EGFR might reflect a role for pUL135 in stimulating EGFR turnover. We examined total cellular levels of EGFR in the context of infection with WT, *UL135*_STOP_ or *UL138*_STOP_ (Fig 2D and 2E). All three viral infections showed a statistically significant decrease in the total EGFR relative to the mock control, reflecting the infection-mediated transcriptional downregulation of EGFR [19]. Relative to mock infection, WT and *UL138*_*STOP*_ infection decreased EGFR levels by 70–75% (p-value≤0.001), with neither being statistically different from each other. In *UL135*_STOP_ infection, total EGFR levels were 50% (p-value≤0.001) reduced relative to mock-infected cells, but 50% increased relative to WT infection (p-value = 0.0013). These results suggest that UL135 stimulates the turnover of EGFR during CMV infection, while UL138 has no affect on total EGFR levels.

To assess whether or not *UL135* turnover of EGFR might represent global modulation of receptor degradation, we also analyzed two other RTKs, platelet-derived growth factor receptor α (PDGFRα) and vesicular endothelial growth factor receptor 2 (VEGFR2), as well as the serine-threonine kinase transforming growth factor β receptor 1 (TGFβR1). We chose PDGFRα and VEGFR2 because they activate similar downstream signaling pathways to EGFR, while TGFβR1 was chosen because it is trafficked similarly to EGFR [20, 21]. As previously published [22], PDGFRα and VEGFR2 were down regulated during WT infection (p-value ≤0.01 for all infections), but their levels are not affected by *UL135* or *UL138* (Fig 2D and 2E). Therefore, while CMV infection affects multiple RTKs during infection, UL135 and UL138 appear to have some specificity for EGFR. TGFβR1 levels were largely unaffected by infection and not affected by either UL138 or UL135. These results suggest some specificity of pUL135 and pUL138 to EGFR.

### pUL135 and pUL138 differentially modulate EGFR trafficking

**Fig 3:**
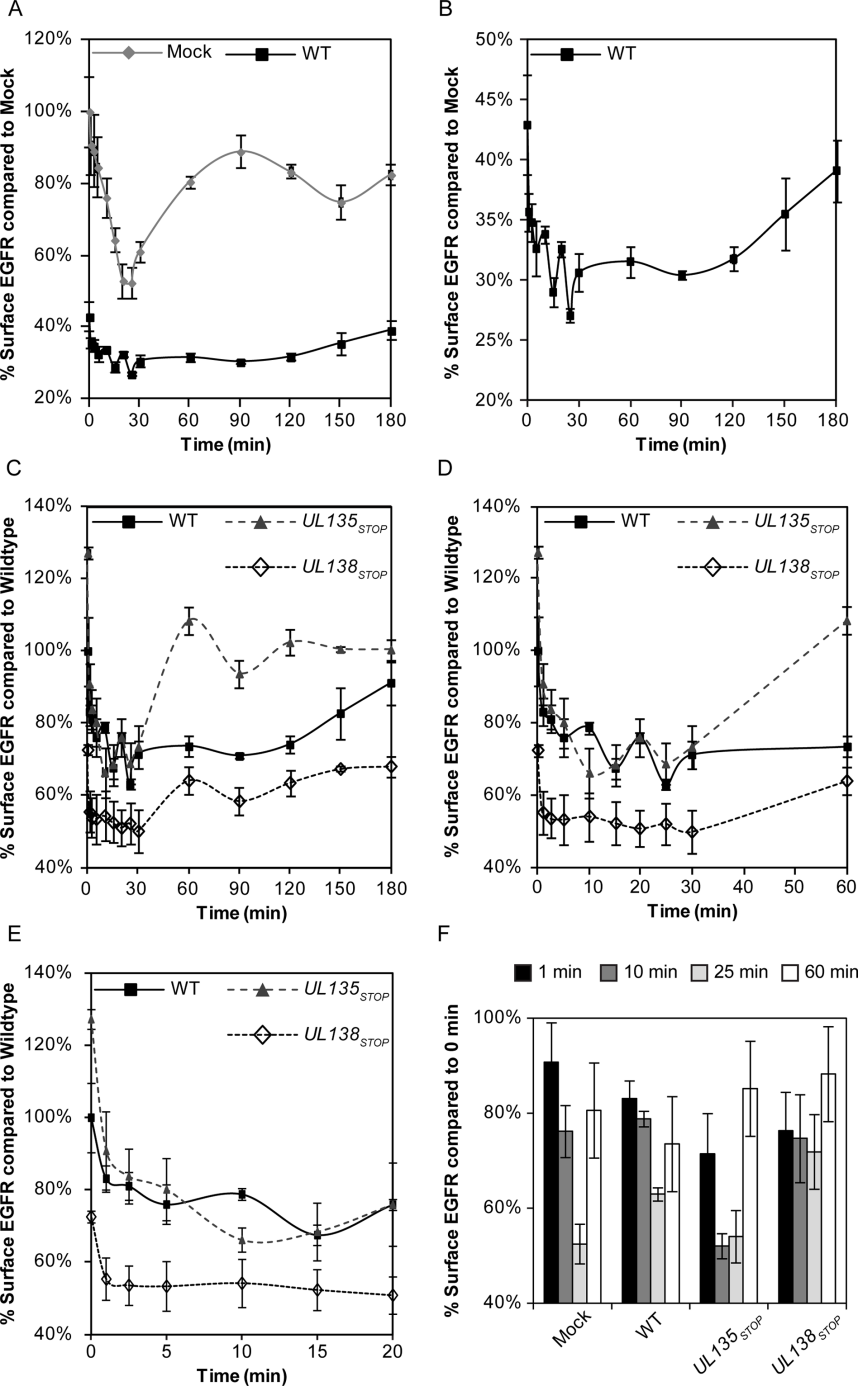
pUL135 and pUL138 regulate EGFR trafficking. Fibroblasts were infected with WT, *UL135*_STOP_, and *UL138*_*STOP*_ virus at an MOI of 1. At 48hpi, cell were stimulated with EGF, collected over a time course of 0–180 minutes post pulse, and stained with BV421 conjugated ms α-EGFR. EGFR surface levels were measured in infected (GFP+) cells by FACS. (A and B) EGFR surface levels over the time course in mock- and WT-infected cells. The WT curve is replotted in panel B on a scale to better discern trafficking dynamics. (C-E) EGFR trafficking during *UL135*_*STOP*_ and *UL138*_*STOP*_ infection in fibroblasts relative to WT. Panel D expands the 0–60 minute time points and panel E expands 0–20 min. (F) Data from selected timepoints were chosen and compared to initial EGFR levels summarize the differences within each infection.

Changes to EGFR surface levels during infection may reflect functions of pUL135 and pUL138 in altering the internalization or recycling of EGFR-containing vesicles. To investigate these possibilities, we monitored changes in EGFR surface levels over time following an EGF pulse. As expected, uninfected cells rapidly internalized EGFR following stimulation with EGF; EGFR reached the lowest surface levels, approximately 52% of the initial levels (zero minute time point), by 25 minutes (Fig 3A). Approximately 90% of the initial EGFR surface levels were recovered by 90 minutes. As would be expected from our findings in Fig 2A, EGFR surface levels were decreased at the zero time point during WT virus infection. Further, EGFR trafficking was severely diminished in WT-infected cells (Fig 3A). The WT-infected cell data is shown on an expanded scale in Fig 3B to better illustrate the trafficking pattern of EGFR. In WT-infected cells, EGFR was maximally internalized by 25–30 minutes (63% of initial levels) post pulse. In contrast to uninfected cells, EGFR internalization was accompanied by oscillation in surface levels between 1 and 30 minutes. The oscillation observed between 10 and 30 minutes in WT or *UL135*_STOP_ infection is a point for further investigation, but may reflect a destabilization of EGFR internalization, rapid recycling back to the cell surface [23], or rapid trafficking of an internal pool of EGFR. Maximal restoration of EGFR surface levels was not observed until 120 minutes post stimulation in WT infected cells. While the increased surface levels at 120 minutes might reflect delayed recycling, this interpretation is confounded by the possibility that at least some portion of the surface levels at this time are contributed by new synthesis of EGFR. These results indicate that CMV infection alters internalization and recycling of EGFR.

To determine the contribution of *UL135* and *UL138* to EGFR trafficking during infection, we compared *UL135*_STOP_ or *UL138*_STOP_ infection to WT infection (Fig 3C, expanded in 3D and 3E). Prior to the EGF pulse, EGFR surface levels were increased or decreased in *UL135*_STOP_ or *UL138*_STOP_ infection relative to that of WT-infected cells, as anticipated from our analysis of surface levels (Fig 2B). The internalization of EGFR following EGF stimulation in *UL135*_*STOP*_ infection reflected that of WT infection at the early time points (Fig 3C–3E), marked by early oscillation in EGFR surface levels. EGFR levels reached their lowest levels by 10 minutes (52% of initial levels). However, unlike WT infection, EGFR surface levels were restored to 85% of initial levels by 60 minutes in *UL135*_STOP_ infection. The kinetics of EGFR trafficking back to the surface during *UL135*_*STOP*_ infection exceeded the kinetics observed in uninfected cells (60 vs. 90 min). In *UL138*_STOP_ infection, maximal internalization was achieved by 1 minute and only 24% of the initial levels were internalized. Notably, the oscillation of surface EGFR observed in WT and *UL135*_STOP_ infection (Fig 3C) was lost in the absence of *UL138*. EGFR levels did not vary more than 2% between 1 to 30 minutes post EGF pulse (Fig 3C; 0–60 minutes expanded in Fig 3D and 3E). Eighty-eight percent of the EGFR surface levels were restored by 60 minutes (Fig 3C–3E). These studies reveal distinct roles for pUL135 and pUL138 in modulating EGFR trafficking. The differences in EGFR trafficking are summarized by plotting EGFR surface levels (relative to levels prior to EGF stimulation in each infection) at 1, 10, 25 and 60 minutes (Fig 3F). We conclude that (i) EGFR trafficking is impeded by CMV infection, (ii) UL135 impedes recovery of EGFR at the cell surface, and (iii) pUL138 impedes internalization or stimulates rapid recovery of EGFR to the cell surface during early times post EGF. These data further support a role for pUL135 in stimulating turnover of EGFR, while revealing a role for pUL138 in modulating EGFR recycling or trafficking to the cell surface from early endosomes.

### pUL135 and pUL138 differentially modulate activation of EGFR

**Fig 4:**
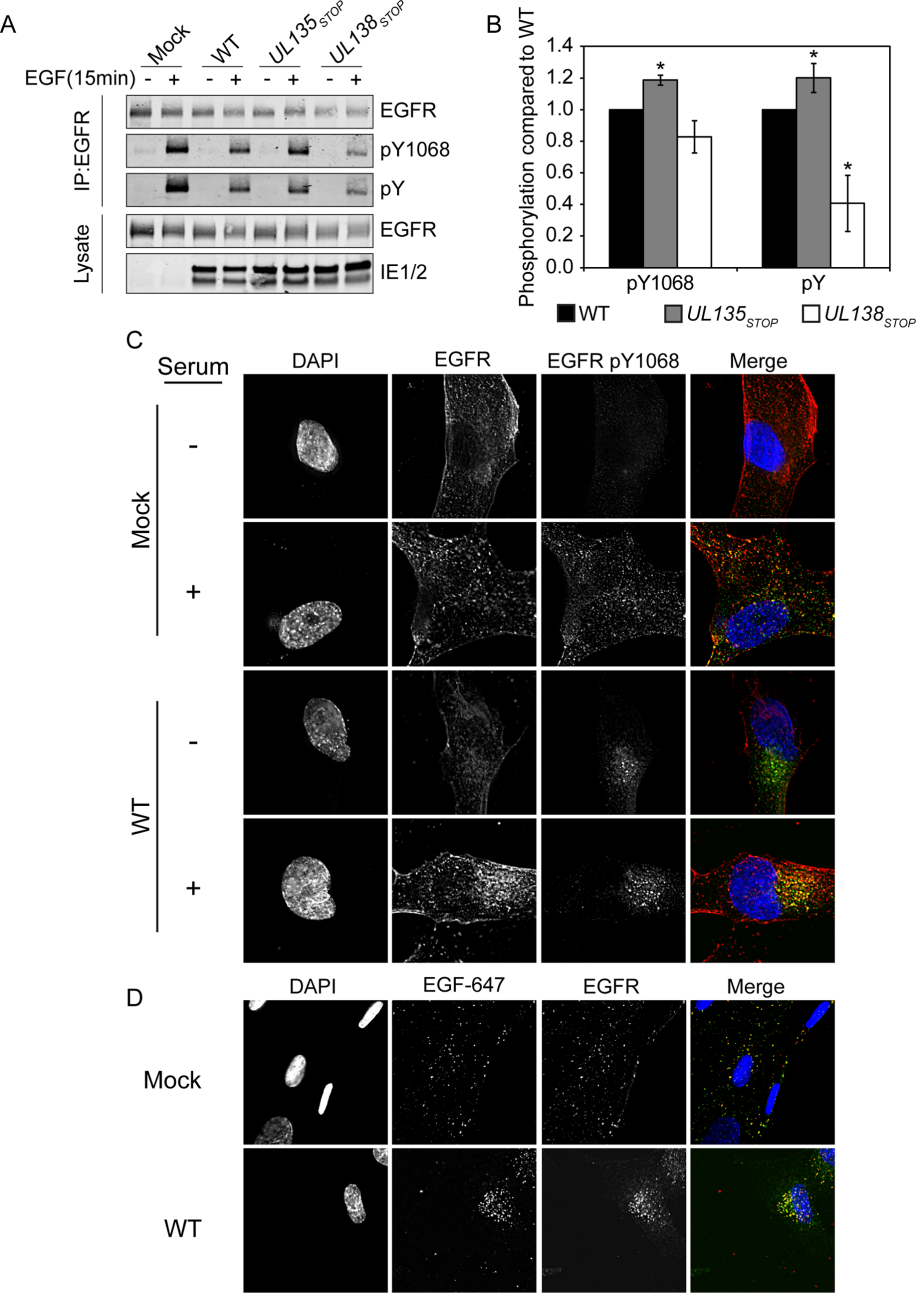
pUL135 and pUL138 impact phosphorylation of EGFR. Fibroblasts were infected with WT, *UL135*_STOP_, and *UL138*_*STOP*_ virus at an MOI of 1. At 48 hpi, infected cells were pulsed with 10nM EGF for 15min and then lysed. (A) EGFR was immunoprecipitated with ms α-EGFR and both IP and lysate samples were separated by SDS-PAGE. Blots were stained with rb α-EGFR, rb α-EGFR phosphotyrosine 1068, ms α-phosphotyrosine, and ms α-IE1/2. (B) The quantification of phosphorylation over three experiments is shown. To control for the variation in EGFR levels in different infections, we normalized signals associated with pY1068 or pY to total EGFR. Statistical significance relative to WT was calculated by student t-test (* p-value ≤ 0.05). (C) Serum-starved or fed fibroblasts expressing EGFR_3XFLAG_ were infected with WT CMV at 20 hours post transduction. Cells were stained with ms α-EGFR, rb α-EGFR pY1068 at 48 hpi. A merge of all three images in shown to the right. (D) Serum-starved, infected fibroblasts were pulsed with Alexa Fluor 647-conjugated EGF ligand on ice, fixed 20 min after a shift to 37C and stained with rb α-EGFR. Cells were imaged by deconvolution microscopy. For C and D, nuclei are stained with DAPI.

Ligand binding induces homo- or heterodimerization of EGFR, and is coupled to the auto- or Src-mediated phosphorylation of a number of sites in the cytosolic tail of EGFR. Autophosphorylation of tyrosine 1068 (Y1068) is an indicator of EGFR activity and is required for binding to the SH2 domain of the growth factor receptor-bound protein 2 (Grb2) [24]. We analyzed phosphorylation of Y1068 following an EGF pulse in the context of infection with or without pUL135 and pUL138 (Fig 4A and 4B). Phosphorylation was induced by EGF stimulation to a similar level in uninfected and WT infected cells when normalized for total EGFR levels, as indicated by pY1068 or pY. However, phosphorylation of Y1068 increased by approximately 20% during *UL135*_STOP_ infection (p-value = 0.002) relative to the WT infection. While not statistically significant, Y1068 phosphorylation tended to decrease in *UL138*_STOP_ infection relative to WT infection. Using the same blots we also analyzed total tyrosine phosphorylation (pY) on EGFR. Again, we detected a 20% increase in pY during *UL135*_*STOP*_ infection (p-value = 0.047). However, during *UL138*_*STOP*_ infection pY staining of EGFR was decreased by 60% (p-value = 0.015), suggesting that pUL138 maintains EGFR signaling during infection, but not necessarily through Y1068 (Fig 4B). These results indicate a role for pUL135 in attenuating EGFR signaling whereas pUL138 functions to maintain it. Further work will be important to determine how pUL135 and pUL138 may affect specific phosphorylation sites on EGFR and how these specifically affect EGFR activity in infection.

### Phosphorylated EGFR accumulates in a juxtanuclear region during CMV infection

Due to the altered trafficking and activation of EGFR, we analyzed the subcellular distribution of EGFR in the context of infection. In uninfected, serum-starved cells EGFR is predominantly localized to the cell surface in an inactive state. Accordingly, phosphorylation of Y1068 is low in these cells (Fig 4C, top row). The addition of serum-containing media stimulated the phosphorylation of EGFR and its localization into cytoplasmic vesicles (Fig 4C, second row). Strikingly, EGFR was predominantly localized to a juxtanuclear compartment in both serum-starved and fed infected cells (Fig 4C, bottom 2 rows). Activated EGFR (pY1068) was detected predominantly in the juxtanuclear compartment irrespective of the serum-starved or–fed state, suggesting that viral infection sequesters and sustains EGFR activity even under serum stress.

We next wanted to determine if the activated EGFR present at the juxtanuclear compartment in infected cells represented EGFR sequestered following its synthesis or trafficked from the cell surface. We labeled serum-starved fibroblasts with EGF ligand conjugated to Alexa Fluor-647 (EGF-647). Twenty-minutes following a temperature shift, internalization of EGF-647 and EGFR were detected in uninfected and infected cells (Fig 4D). However, in infected cells, EGF-647 and EGFR were localized to the juxtanuclear compartment, indicating that the juxtanuclear EGFR is trafficked from the cell surface.

### EGFR is sequestered in the recycling endocytic vesicles in the viral assembly compartment

**Fig 5:**
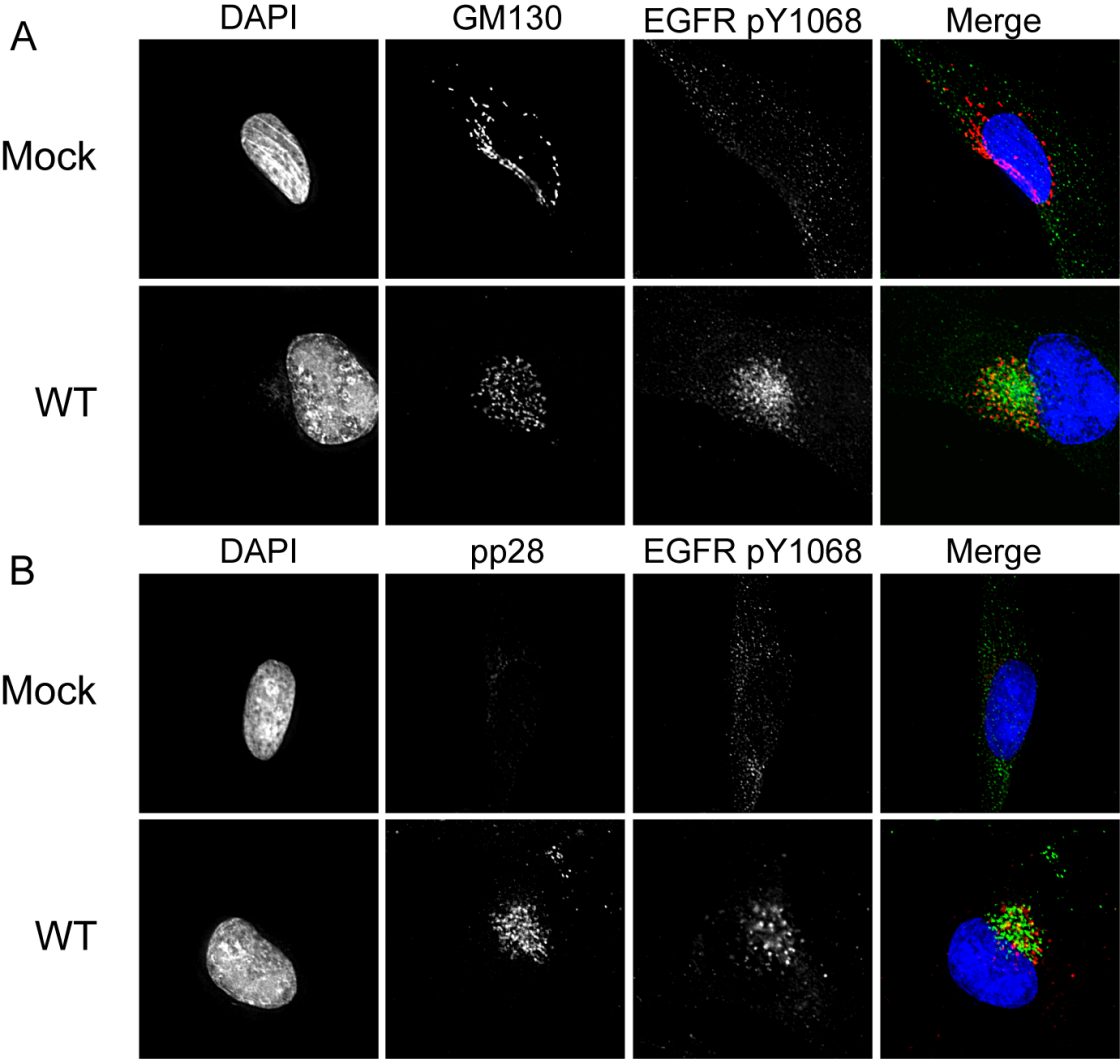
Activated EGFR localizes to the viral assembly compartment irrespective of stimulation. Mock- or WT-infected fibroblasts were stained with rb α-EGFR pY1068 and (A) ms α-GM130 to stain the Golgi or (B) ms α-pp28 as a marker for the viral assembly compartment. Cells were imaged by deconvolution microscopy. For all panels, nuclei are stained with DAPI. A merge of all three images in shown to the right.

The juxtanuclear localization of EGFR resembles the viral assembly compartment (VAC), a virus-induced reorganization of endo- and exocytic membranes that functions in capsid tegumentation and envelopment [25–27]. We sought to determine if EGFR was localized to the VAC and define the EGFR-containing vesicles. We analyzed Y1068 localization with the cis-Golgi marker, GM130 (Fig 5A), and the viral tegument protein, pp28 (Fig 5B), both established markers for the VAC. EGFR localized in the region with GM130 and pp28, although the staining was not co-incident. We have previously reported that pUL135 and pUL138 also localize to the VAC during infection [4, 8]; however, we did not observe any difference in the localization of pY1068 EGFR between WT, *UL135*_STOP_ or *UL138*_STOP_ infections (S2 Fig).

### EGFR association with Rab5 and Rab11 vesicles increases in infection

**Fig 6:**
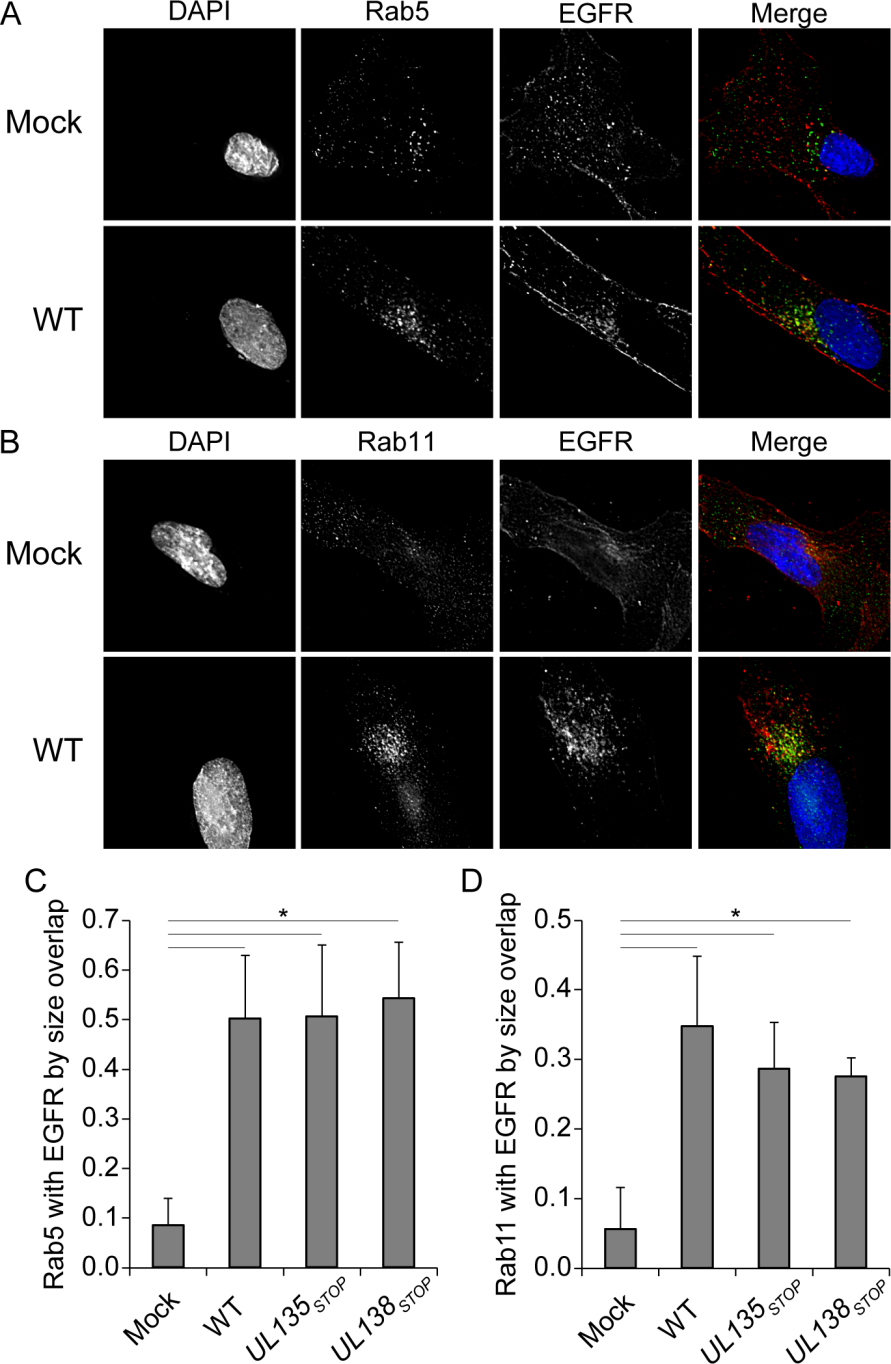
EGFR association with Rab 5 and Rab 11 vesicles increased in the context of infection. Fibroblasts over-expressing EGFR_3XFLAG_ were stained with ms α-EGFR and (A) rb α-Rab5 as a marker of sorting endosomes, or (B) rb α-Rab11 to stain the ERC at 48 hpi. Cells were imaged by deconvolution microscopy. For all panels, nuclei are stained with DAPI. A merge of all three images in shown to the right. The extent of colocalization between (C) Rab 5 or (D) Rab 11 vesicles was quantitated by the Sqaussh.workfkow in the Mosaic suite of Image J and Fiji. The y axes represent the average proportion of the indicated Rab that is coincident with EGFR. Statistical significance was determined by a one-way ANOVA with Tukey correction for percent overlap. Standard deviation is depicted by error bars. Asterisks represent statistically significant differences (p values <0.001).

To define the EGFR-containing vesicles in the VAC, we analyzed the co-localization of EGFR with a number of Rab proteins that serve as endocytic vesicle markers. Rab proteins are small GTPases that modulate distinct membrane trafficking events. Rab 5 is localized to sorting endosomes and mediates fusion of early and late endosomes [28]. Rab11 marks the endocytic recycling compartment (ERC) and the trans-Golgi network and is involved in late endocytic recycling events. Typically, the association of EGFR with Rab 5 vesicles is transient and not observed at steady state, consistent with our findings in uninfected cells (Fig 6A and 6C). EGFR is not typically sorted to the Rab 11-positive ERC under normal growth, but, EGFR has been observed to recycle in Rab 11 vesicles in states of stress, drug treatment or in immortalized cells [29, 30]. Accordingly, the colocalization of EGFR with Rab 11 in uninfected cells was minimal (Fig 6B and 6D). However, the association of Rab5 (Fig 6A and 6C) and Rab11 (Fig 6B and 6D) with EGFR was increased in the context of CMV infection. The extent of Rab 5 or Rab 11 co-localization with EGFR was quantitated by two methods: Rab coincidence with EGFR and Pearsons correlations using the Image J Mosaic suite Squassh workflow [31, 32]. While there was a significant increase in the association of Rab 5 or 11 and EGFR between uninfected and infected cells (Fig 6C and 6D), we did not observe a statistically significant change between WT and mutant virus infections. Similar results were obtained with Pearson correlations. The coincidence of Rab 5 or Rab 11 with cytosolic EGFR has a Pearson correlation of 0.1 in uninfected cells, which rose to ≥0.3 in all infection conditions analyzed. Defining differences in vesicle association between WT and mutant viruses will likely require dynamic assays that follow a pulse of EGF over time, similar to those in Fig 3. The discrete association of EGFR with Rab5 and Rab11 vesicles in the context of infection suggests that CMV induces the accumulation of EGFR in vesicles poised for its recycling during CMV infection.

### EGFR signaling suppresses viral replication in fibroblasts

**Fig 7:**
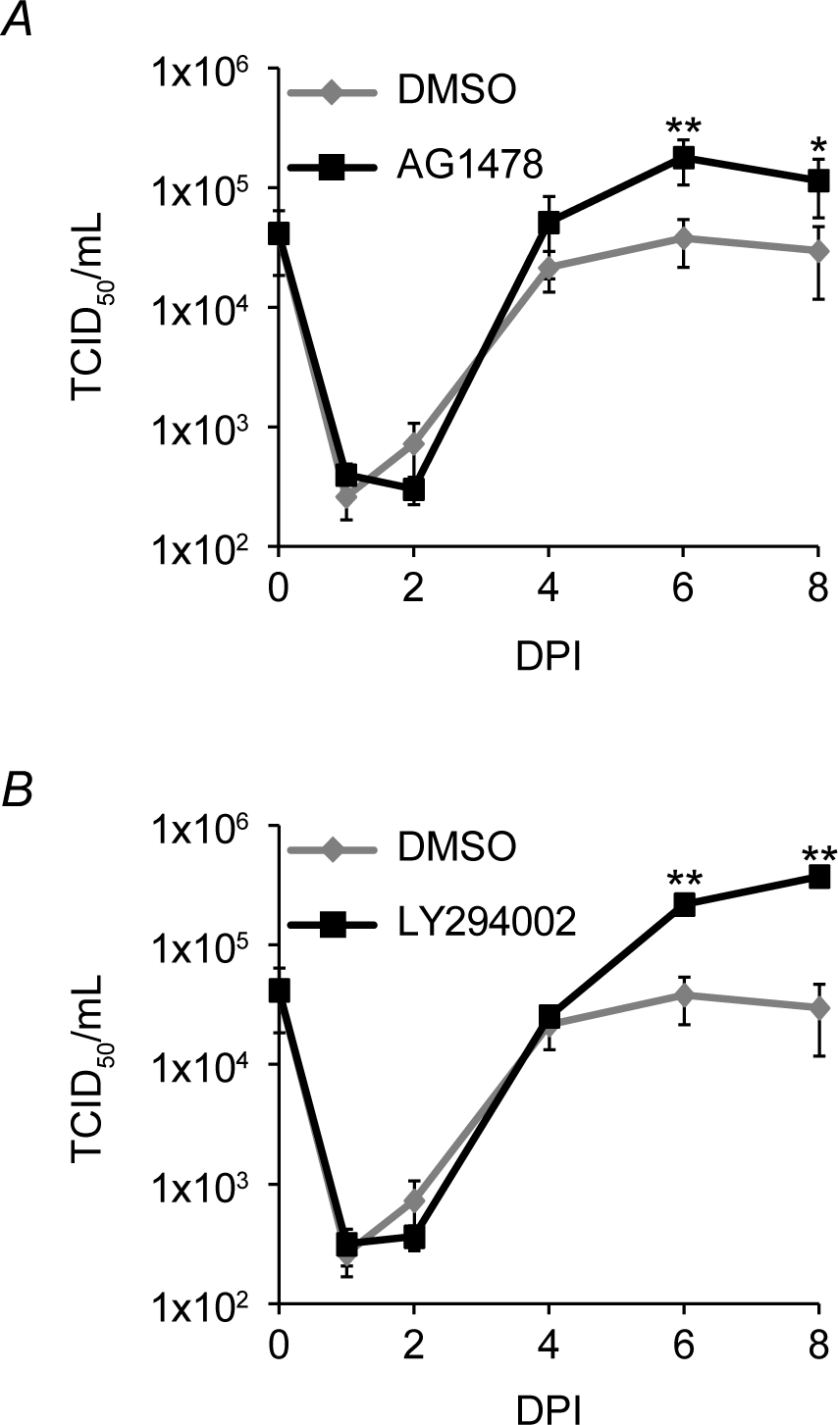
Inhibition of EGFR signal promotes viral replication. Fibroblasts infected with 0.5 MOI of WT virus were treated with DMSO, (A) 5 μM AG1478 or (B) 20 μM LY294002 at 18hpi. Cells and media were collected over a time course and virus yields were determined by TCID_50_. Values represent the average of three independent experiments with SEM shown. Statistical significance was determined by two-way ANOVA with Bonferroni correction for differences between vehicle controls and drug treatments are indicated by asterisks (* p-value<0.05; ** p-value<0.01).

While EGFR and PI3K activation has been shown to be important for entry of HCMV into fibroblasts and monocytes [33–35], nothing is know about the role of EGFR throughout infection. Based on our observation that *UL135*, an activator of replication, induced the turnover of EGFR and EGFR is transcriptionally downregulated during replication in fibroblasts [18, 19], we hypothesized that reduced EGFR levels and activity in the context of viral infection promoted virus replication. To determine a role for EGFR and its downstream phosphatidylinositol 3-kinase (PI3K) signaling in virus replication, we analyzed CMV replication over time in the presence or absence of the EGFR kinase inhibitor, AG1478 (Fig 7A) or the PI3K inhibitor LY294002 (Fig 7B). So as not to interfere with viral entry, inhibitors were not applied to cells until after viral entry. Inhibition of EGFR increased replication in fibroblasts by 5-fold at 6 dpi (p-value<0.01) and maintained a statistically significant increase at 8 dpi (p-value<0.05) relative to vehicle control (Fig 7A). The PI3K inhibitor enhanced replication by 6-fold (Fig 7B) at 6 dpi (p-value<0.01) and 13-fold by at 8 dpi (p-value<0.01). These data demonstrate that inhibition of EGFR and PI3K activity enhances CMV replication.

### Inhibition of EGFR promotes reactivation from latency

Loss of *UL138* results in a virus that is unable to establish or maintain a latent infection in CD34^+^ HPCs and instead replicates productively. *UL135* functions, in part, to overcome *UL138*-mediated suppression and loss of *UL135* results in a failure to reactivate from latency [10]. These findings demonstrate an antagonism between *UL138* and *UL135* that governs entry into and exit from latency. Given the antagonistic regulation of EGFR by pUL135 and pUL138 defined here, we hypothesized that the opposing functions of pUL138 and pUL135 in regulating EGFR impacts states of latency and reactivation in CD34^+^ HPCs. As surface expression of EGFR is not well established in CD34+ HPCs, we first investigated EGFR surface levels in the context of WT, *UL135*_STOP_ and *UL138*_STOP_ virus infection using EGF-647. We verified the specificity and sensitivity of this assay using human embryonic kidney-293 cells, which express little to no EGFR [23]. HEK-293 cells transfected with a plasmid expressing EGFR and labeled with EGF-647 increased fluorescent signal 2500-fold compared to cells not expressing EGFR (S3A Fig). In contrast, a fluorescently-conjugated EGFR antibody increased staining by only 800-fold relative to the control, indicating increased sensitivity of EGF-647 ligand for detecting surface levels of EGFR. In CD34^+^ HPCs, detection of EGFR at the cell surface was similarly enhanced 5-fold by EGF-647 ligand (S3B Fig), while the fluorescently conjugated antibody to EGFR was unable to detect EGFR on the surface of CD34^+^ HPCs.

**Fig 8:**
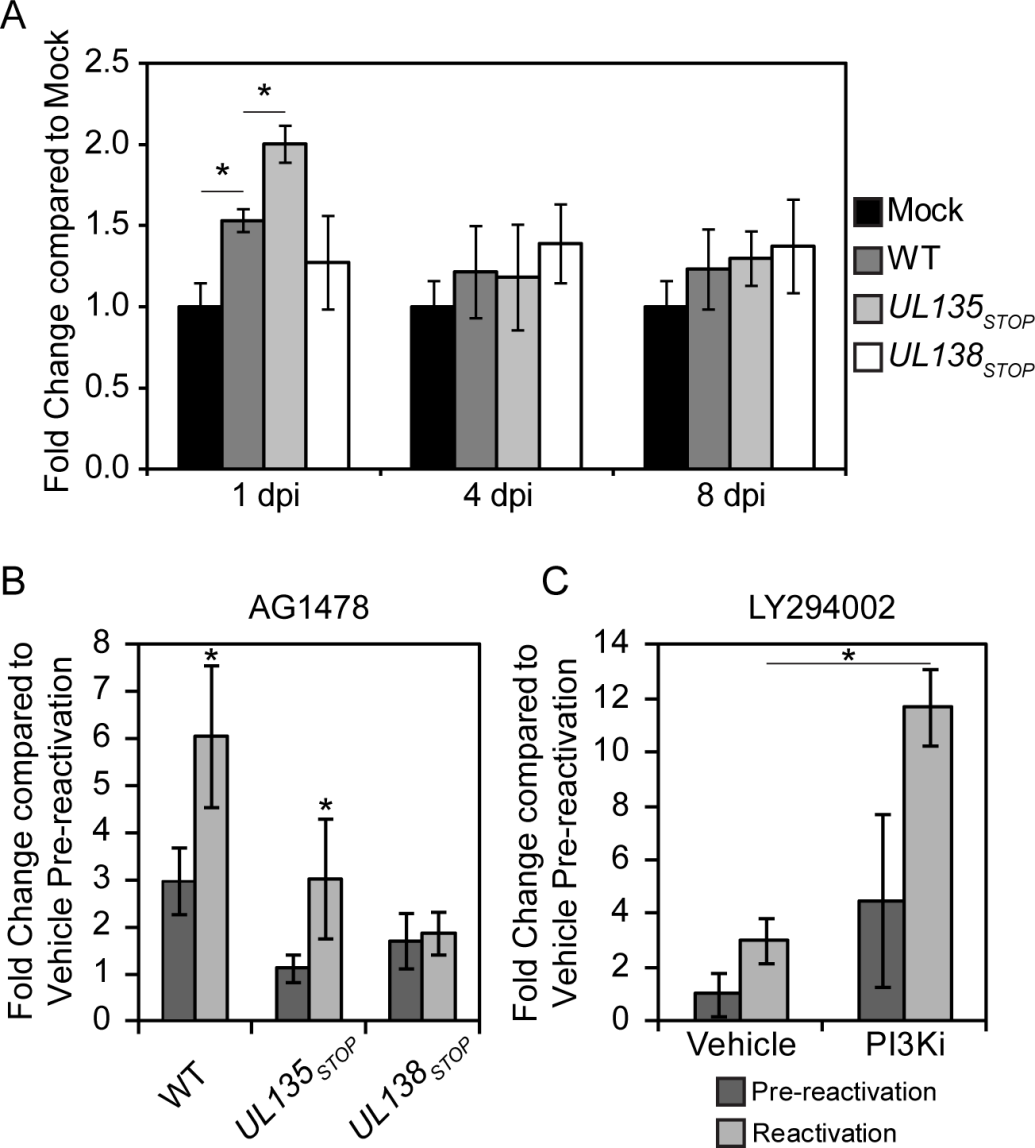
EGFR signaling maintains latency. CD34^+^ HPCs were infected with WT, *UL135*_*STOP*_, and *UL138*_*STOP*_ virus at an MOI of 2. (A) At 1, 4, and 8 dpi cells were stained with Alexa Fluor 647-conjugated EGF ligand and BV421-conjugated ms α-CD34 for FACS analysis to measure EGFR surface levels. Bars represent the average of three independent experiments where EGFR surface levels are relative to mock infection. Standard error of the mean is shown. Asterisk indicates significance by Student’s t-test where p<0.05. (B) Pure populations of CD34^+^ HPCs infected with WT, *UL135*_STOP_, or *UL138*_STOP_ at an MOI of 2 were isolated by FACS at 24 hpi and maintained in LTBMC with EGFR inhibitor AG1478 at 10 μM. At 10 dpi, viable CD34^+^ HPCs were seeded by limiting dilution onto monolayers of permissive fibroblasts (reactivation). An equivalent number of cells were mechanically disrupted and seeded in parallel to determine the infectious virus present in the culture prior to reactivation (pre-reactivation). Frequency of infectious centers formed pre and post reactivation was determined 14 days later from the number of GFP-positive wells at each dilution using ELDA software. Bars represent the average fold change in drug-treated cells relative to vehicle (DMSO) pre-reactivation control for three independent experiments. Standard error of the mean is shown. (C) WT-infected CD34+ cells treated with PI3K inhibitor LY294002 at 20 μM were analyzed as described for panel B. (B-C) Significance determined by two-way ANOVA with Bonferroni correction for differences between vehicle controls and drug treatments, asterisk indicates p-value<0.05.

In contrast to infection in fibroblasts (Fig 2), EGFR surface levels were increased 1 dpi in WT-infected CD34+ HPCs relative to uninfected CD34^+^ HPCs (Fig 8A). *UL135*_*STOP*_ infection resulted in increased levels of EGFR relative to WT infection, consistent with our findings in fibroblasts. Surface levels of EGFR in *UL138*_*STOP*_ did not change significantly relative to WT infection on uninfected cells. The alterations in surface expression of EGFR were transient and were not observed at 4 and 8 dpi time points. These results suggest that pUL135 and pUL138 alter surface EGFR expression early in infection of CD34^+^ HPCs.

To determine if EGFR activity mpacts CMV latency or reactivation from latency, we chemically inhibited EGFR signaling in infected CD34^+^ HPCs. Pure populations of infected (GFP^+^) CD34^+^ HPCs were seeded into long-term bone marrow culture in the presence of the AG1478 EGFR inhibitor or a DMSO vehicle control. After 10 days in culture, the cells or an equivalent cell lysate were seeded by limiting dilution in a cytokine-rich media to promote reactivation on monolayers of permissive fibroblasts. The infectious centers detected in the cell lysate reflect the virus produced prior to reactivation and serves as a “pre-reactivation” control. The frequency of infectious centers produced in CD34+ HPCs treated with AG1478 are represented as the fold change in infectious centers relative to the cells treated with vehicle control (Fig 8B). Inhibition of EGFR in WT infection resulted in a 3-fold and 6-fold greater frequencies of infectious centers produced prior to reactivation and following reactivation, respectively. While EGFR inhibition did not enhance *UL135*_STOP_ replication in unstimulated CD34^+^ HPCs (Pre-reactivation), inhibition enhanced the frequency of reactivation of *UL135*_STOP_ by 3-fold. This result indicates that the inhibition of EGFR in combination with a reactivation stimulus partially complements the defect in reactivation of the *UL135*_STOP_ virus [10]. In the case of *UL138*_*STOP*_, which replicates in the absence of a reactivation stimulus, inhibition of EGFR did not significantly increase infectious centers formation either prior to or following reactivation. Similar to AG1478, another EGFR kinase inhibitor, Gefitinib, increased the frequency of infectious centers formation in WT and *UL135*_STOP_ infection, but not *UL138*_STOP_ (S4 Fig). We also analyzed the effect of PI3K inhibition on the WT infection in CD34^+^ HPCs. Treatment of cells with PI3K inhibitor LY294002 increased infectious centers production in WT infection 3-fold in combination with a reactivation stimulus (Fig 8C). While the PI3K enhanced infectious centers formation in unstimulated cells 2-fold, this difference was not statistically significant. From these results we conclude that the inhibition of EGFR or downstream PI3K enhances reactivation in CD34^+^ HPCs. These results reveal a role for EGFR and PI3K in maintaining latency during CMV infection in CD34^+^ HPCs.

## Discussion

Herpesviruses have evolved complex interactions with the host to achieve lifelong persistence. To avoid elimination, herpesviruses masterfully evade intrinsic, innate and adaptive defenses to infection. Although less well defined, herpesviruses also modulate epigenetic silencing and homeostatic signaling in the host cell to create an optimal environment for persistence. EGFR is a major homeostatic regulator of cell proliferation, differentiation, adhesion/migration, survival [36, 37], and most recently, innate signaling [38, 39] and DNA repair [40]. As such, EGFR represents a potentially powerful target for viral manipulation during infection with complex DNA viruses.

Many viruses target EGFR: RNA viruses (e.g., rhinovirus, RSV, influenza and measles) induce EGFR [41–43], while DNA viruses (e.g., adenovirus, CMV and HSV) typically inhibit EGFR [19, 44–46] during their replicative cycles. EGFR has been reported to be an entry receptor for CMV [33]. In the context of HCMV replication, EGFR is transcriptionally downregulated at early times during a productive infection due to the induction of Wilms’ Tumor Factor 1 [18, 19], a known transcriptional repressor of EGFR. As a result, CMV infection decreases the responsiveness of infected fibroblasts to external stimulation [18]. These observations were made using laboratory strains of CMV, indicating that UL*b*’ region genes are not required for the transcriptional downregulation of EGFR. EGFR signaling has also been shown to be critical for viral entry into monocytes and for monocyte survival and migration [34, 47]. In the context of infection in endothelial cells, a proposed site of CMV persistence, CMV binding to EGFR and β1 and β2 integrins induces increased EC proliferation, motility and capillary tube formation indicative of an angiogenic response [48]. Taken together, these studies implicate EGFR as a major host regulator of infection contributing to CMV persistence in broad contexts of infection. The mechanisms by which and to what end viruses target EGFR are largely undefined.

**Fig 9:**
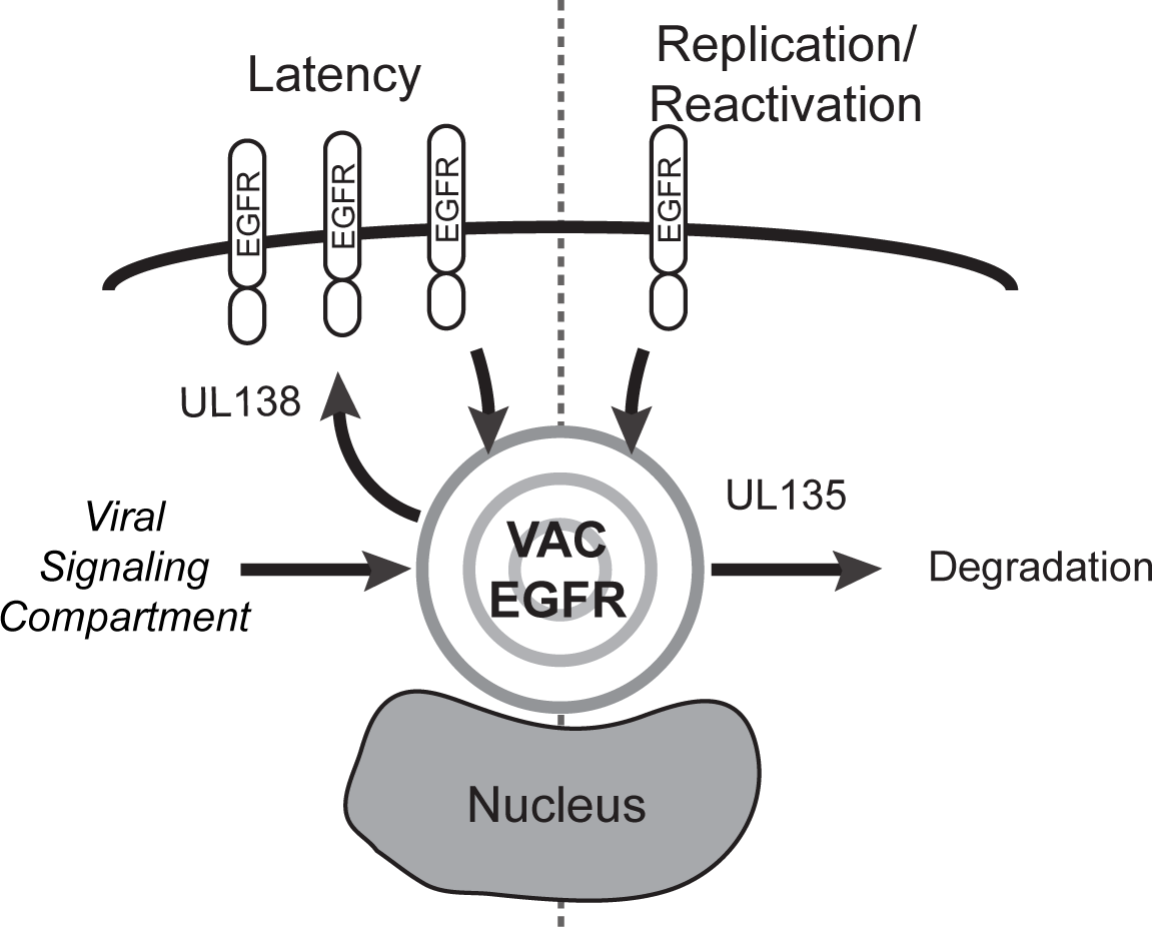
A model for pUL135/pUL138-mediated regulation of EGFR trafficking. We hypothesize that pUL135 and pUL138 oppose one another in the regulation of EGFR to affect replicative or latent states of infection, respectively. pUL135 impedes recycling of EGFR back to the cell surface or promotes its turnover, whereas pUL138 sustains EGFR surface levels and activity. Within productively infected cells, EGFR is associated with endocytic vesicles localized to the VAC. As mTOR and transferrin have also been shown to localize in the VAC, we further propose that the VAC may function as a “viral signaling compartment” in addition to its role in virus maturation. This compartment may allow HCMV to insulate host signaling from extracellular up or downregulation or be a means by which the virus sense and responds to host cues.

Our study defines EGFR as a target of viral manipulation that impacts CMV latency (Fig 9). Further, we have identified two antagonistic viral proteins, pUL135 and pUL138, that target EGFR with opposing effects. The opposing regulation of a single host target by two viral proteins defines a molecular switch to allow the virus to sense and respond to changes in the cellular environment. While likely an over simplification, pUL138 promotes latency by maintaining EGFR surface levels and activity in infected cells, while pUL135 mediates the turnover of EGFR from the cell surface for viral replication and reactivation (Fig 9). This opposing regulation may result in a more sensitized state in CD34^+^ HPCs that is poised to respond to changes in the host, while EGFR signaling in fibroblasts is more insulated. Heightened EGFR levels at the cell surface may contribute to the establishment of latency by modulating cell survival, differentiation or innate signaling. Sustained levels of EGFR may be less important in the context of virus replication because the virus expresses genes to prevent cell death, evade the immune response, or promote protein synthesis.

The regulation of EGFR during CMV infection likely reflects a fine-tuning of host signaling, not an all or none event. EGFR activates several major downstream pathways, including mitogen-activated protein kinase (MAPK)/ extracellular signal-regulated kinase (ERK), PI3K, phospholipase C gamma/protein kinase C (PLCγ/PKC), and signal transducer and activator of transcription (STAT). Some activities downstream of EGFR are required for replication and reactivation, including that of the mammalian target of rapamycin, mTOR [49–51]. Therefore, it is possible that CMV suppresses EGFR for reactivation, while selectively maintaining activity of specific downstream pathways, such as mTOR. More work is required to understand how CMV regulates specific activities of EGFR and its downstream pathways and how this regulation impacts latency and reactivation.

Our studies demonstrate that EGFR activity and that of downstream PI3K activity negatively impact virus yields in fibroblasts (Fig 7) and reactivation in CD34^+^ HPCs (Fig 8). In apparent contradiction, PI3K was reported to promote HCMV replication in fibroblasts [35]. However, cells were pre-treated with PI3K inhibitors prior to infection and the inhibitor was replenished throughout infection in these studies. Therefore, the requirement of PI3K for CMV replication reported in these studies cannot be separated from its requirement for viral entry [33], which was discovered years after these initial studies. These studies also used laboratory strains of HCMV at very high MOI, which may also impact the apparent differences between our findings. Future work will define the specific aspects EGFR function that benefit viral latency or hinder virus replication.

Localization of activated EGFR to the VAC was an unexpected result (Figs 4C and 4D and 5A and 5B). The VAC has been considered a primary site of virion maturation where the virion acquires a full complement of tegument and an envelope [25–27]. Further, the presence of active EGFR at the VAC even when cells were under the stress of a starved state suggests that CMV insulates EGFR from host feedback mechanisms. Consistent with our findings, CMV infection has previously been shown to circumvent cellular stress responses, such as amino acid deprivation, and relocalize activated mTOR to the VAC [52]. Transferrin has also been shown to localize within the VAC during infection [25, 53] and its recycling has been shown to be suppressed by CMV microRNAs targeting host secretory factors [54]. Sequestration of host signaling factors in the VAC suggests a role for the VAC as a “viral signaling compartment” that insulates host signaling molecules or sustain virus-prescribed levels of signaling (Fig 9). pUL135- and pUL138-mediated control of EGFR may underlie the ability of the virus to sense and respond to changes in extracellular environment. It will be interesting to understand how this putative role of the VAC relates to the maturation of virus particles. While the VAC has not been studied in the context of latency in CD34^+^ HPCs, we have described roles of pUL135 and pUL136 in regulating formation of the VAC and viral maturation in endothelial cells, a site of chronic infection in the host [7]. The defects in VAC formation and virion maturation in the absence of *UL135* or *UL136* expression may reflect a primary role of these proteins in modulating membrane and protein trafficking in the host to manipulate cellular signaling and homeostasis, and in turn affect the outcome of infection.

The VAC is comprised of many host secretory membranes, including those important to endocytic sorting and recycling [25, 53]. Consistent with our findings, previous studies have shown that Rab11 and transferrin localize to the VAC [25, 53] and that Rab5 is partially localized to the VAC [55]. The steady state association of EGFR with Rab5 and Rab11 vesicles in the context of infection (Fig 6) was surprising and indicates that HCMV infection has altered host trafficking. In uninfected cells, EGFR transiently passes through Rab5 vesicles, but is not known to traffic to the Rab11-marked ERC except under exceptional conditions of cellular stress associated with drug treatment, cancer or immortalizaion [28–30]. EGFR is commonly used a marker for trafficking through an early endosomal/lysosomal route exclusive of the ERC. Therefore, the increased association of EGFR with Rab 5 vesicles may indicate an increased transit time through this compartment and the induced association with Rab11 vesicles may indicate a redirection of trafficking that reflects a state of stress. In studies exploring the ERC in infection, Britt and colleagues reported that EGF did not localize to the VAC [53]. While the reason for the discrepancy between these findings and the current study is not known, one intriguing possibility is that UL/b’ proteins are required for EGFR localization to the VAC. While we have not observed a role for pUL135 or pUL138 in trafficking EGFR to the VAC (S2 Fig), other ULb’-coded proteins may be important for re-orienting trafficking in the cell. Another possibility is that localization of EGF ligand to the VAC is transient. Britt and colleagues exposed cells to EGF ligand for 2 hours prior to fixation, whereas we examined cells fixed at 20 minutes following exposure to ligand. Therefore, our studies may demonstrate trafficking of EGF to the VAC that then exits the VAC by 2 hours. Kinetic studies will be required to define how CMV infection affects vesicle trafficking.

In addition to its role in regulating surface levels of EGFR in infection, pUL138 has been shown to reduce MRP-1 and enhances TNFR1 levels at the cell surface, although the significance of these changes to latency is not known [13–15]. As TNFα stimulates CMV reactivation [56–58], increased surface levels of TNFR1 may poise cells for reactivation. The mechanism by which pUL138 differentially regulates surface protein expression is not known. In contrast to our findings for EGFR, pUL135 overexpression did not affect surface expression of TNFR1 [13]. Therefore, the partnership between pUL138 and pUL135 in regulating EGFR may not extend to all proteins targeted by pUL138. Moreover, while CMV alters the levels of many proteins [59], the effect of pUL135 and pUL138 on EGFR appears to be specific, as they did not affect total levels of similar RTKs, including PDGFRα and VEGFR2, or a receptor trafficked similarly to EGFR, TGFβR1.

The role of pUL135 reorganization of the actin cytoskeleton and reduced focal adhesion is mediated by an interaction with the Abelson interacting protein-1 (Abi-1), which reduces natural killer cell recognition and killing [17]. Notably, Abi-1 contains Src homology 3 (SH3) domains that mediate its interaction with the Cbl E3 ubiquitin ligase and impact endocytosis and signaling [60]. Intriguingly, Abi1 regulates the phosphorylation and Cbl-mediated turnover of EGFR, EGF-induced Ras, ERK and PI3K pathways [60–62]. The role of the Abi-1-pUL135 interaction in pUL135-mediated turnover of EGFR is not yet defined. pUL135 was also recently demonstrated to contribute to the turnover of the Rho-associated kinase, Rock1, that regulates cytoskeleton [63]. This result suggests that pUL135 functions in targeting other cellular proteins for turnover. A role pUL135-mediated turnover of Rock1 in latency or reactivation has not yet been determined.

Oncomodulatory properties have been attributed to CMV in the case of glioblastoma [64]. EGFR is driver of a number of cancers [37], and is overexpressed in ~54% of glioblastomas [65]. Thus, pUL138-mediated stimulation of surface expression in latently infected cells raises the possibility that viral regulation of EGFR contributes to the oncomodulatory properties of CMV. While the increase in EGFR expression induced by CMV infection of CD34^+^ HPCs *in vitro* was transient (Fig 8A), this may reflect limitation of CD34^+^ culture. Natural oscillations between states of latency and subclinical reactivation in the host might result in heightened or sustained EGFR expression. *UL138* expression is a marker for CMV sero-positivity [11] and, as such, is likely expressed throughout the persistence of the virus.

More broadly, RTKs and PI3K signaling have been shown to play key roles in herpesviral latency. PI3K stimulation through nerve growth factor-binding to the RTK, TrkA, is required for the maintenance of HSV-1 latency in neurons [66]. The HSV-1 tegument protein VP11/12 activates PI3K to stimulate phosphorylation of Akt; however, other as yet unidentified viral proteins are also involved [67]. Similar to the role of pUL138 in stimulating surface expression of EGFR and promoting viral latency, latency membrane protein-1 (LMP-1) of EBV enhances expression and activity of EGFR [68, 69]. LMP-1 activates EGFR, STAT3, and ERK pathways through PKCδ [70], while LMP-2A constitutively activates PI3K and Akt pathways [71, 72] and stabilizes β-catenin [71]. The PI3K/Akt pathway also maintains latency in murine herpesvirus γ-68 replication and Kaposi’s sarcoma-associated herpesvirus (KSHV) infection [73]. The K1 glycoprotein of KSHV activates Akt downstream of PI3K to enhance cell survival [74]. Our work identifying opposing viral regulators of EGFR offers a powerful path towards defining the mechanistic underpinnings and the significance of viral-mediated control of EGFR and its downstream signaling pathways to viral infection.

Understanding the complex interplay between herpesviruses and host signaling at the molecular level is important for defining the mechanistic basis of viral latency and persistence. This work defines a molecular switch that regulates latent and replicative states through the modulation of EGFR. The regulation of EGFR at the cell surface provides a novel means by which the virus may sense and respond to changes in the cellular environment to enter into, maintain or exit the latent state. There is no vaccine for CMV and CMV antiviral therapies are limited in their ability to control CMV because they do not target latently infected cells. Our work identifies potential targets for the design of novel antiviral strategies aimed at latent reservoirs.

## Materials and Methods

### Cells

Primary lung fibroblasts (MRC-5;ATCC), HEK-293T/17 (ATCC), Sl/Sl (Stem Cell Technologies), M2-10B4 (Stem Cell Technology), and CD34+ HPCs were maintained as previously described [75]. Human CD34+ HPCs were isolated, as previously described [75], from de-identified bone marrow and cord blood samples obtained from the University Medical Center at the University of Arizona, in accordance with our Institutional Review Board.

### Viruses

TB40/E WT, *UL135*_*STOP*_¸and *UL138*_*STOP*_ are described in Umashankar et al. 2014a. TB40/E *UL138*_*3xFLAG*_ was described by Petrucelli et al. 2012b. TB40/E *UL133*_*MYC*_ was created by a similar fashion as was previously described for FIX-*UL133*_*MYC*_ [76].

### Plasmids

All Primer sequences can be found in Table 1. pCIG*-UL135*_*MYC*_ is previously described [75]. pCIG-*UL135*_*V5*_ was created by amplifying UL135_V5_ with UL135-FWD and UL135-V5-REV primers. The product was digested with NheI and BamHI, gel purified, and ligated into pCIG to create pCIG-*UL135*_*V5*_. *UL138* was amplified from TB40/E with XbaI-UL138-Fwd and UL138-myc-NheI-Rev primers and ligated between XbaI and NheI digestion sites of pCIG. pCIG-*UL37*_*MYC*_ was created by digesting pcDNA3-*UL37*_*MYC*_ (Victor Goldmacher; Immunogen) with HindIII and XbaI, blunting the *UL37*_*MYC*_ fragment with Klenow, and ligating into the EcoRI site of pCIG. HA-tagged ubiquitin (_HA_Ub) polypeptide sequence was subcloned from pCMV-_HA_Ub into pCIG via NheI and EcoRI sites. EGFR was amplified from pCIG-EGFR_GFP_ using pCIG Fwd and EGFR 3xFlag Rev. EGFR_3xFLAG_ product was then digested with NheI and ligated in pCIG expression vector that was digested with BamHI, blunted with Klenow, and redigested with NheI. Lentiviruses were created by cotransfecting HEK293T/17 cells with pCIG vectors, pMD2.G, and psPAX2 (Addgene #12259 and 12260; Trono Lab) using polyethylenimine (Polysciences) and collecting supernatants 48h later.

**Table 1:** Primer sequences.

Primer	Sequence
UL133-PhuFWD	5’-CCCGCCCCCGGTGTGATAAGGAATTTTCCG-3’
UL133-PhuFWD	5’-[phos]TTACAGATCCTCTTCTGAGATGAGTTTTTGTTCACCGCCACCGCCCGTTCCGG TCTGATGCTGCTGCTG-3’
UL135-FWD	5’-CGCGGATCCGCTAGCACCATGGTGTGGCTGTGGCTCGGCG TCG GGCTCCTCGGC-3’
UL135-V5-Rev	5’-CGCGGATCCTCACGTAGAATCGAGACCGAGGAGAGGGTTAGGGATAGGCTTACCGCT TCCTCCTCCGGTCATCTGCATTGACTCGGCGTCCTTCATGAC-3’
UL138-Fwd	5’-GGGGTCTATAATGGACGATCTGCCGCTGAACGTCGGG-3’
UL138-myc-NheI-Rev	5’- GGGGCTAGCTCACAGATCCTCTTCTGAGATGAGTTTTT GTTCCG-3’
pCIG Fwd	5’- GGGAGGTCTATATAAGCAGAGCTCGTTTA-3’
EGFR 3xFlag Rev	5’-[phos]ATGGATATCTTATTTATCATCATCATCTTTATAATCAATATCATGATCTTTATAAT CGCCATCATGATC'TTTATAATCACCGCCACCGCCTGCTCCAATAAATTCACTGCTTT-3’

### Mass spectrometry

To identify pUL138 interacting partners, fibroblasts were infected at an MOI of 3 with TB40/E-*UL138*_*3XFLAG*_. Interacting proteins were isolated by cryogenic cell lysis at 48hpi and rapid immunoaffinity purification as previously described [77]. Briefly, protein complexes were isolated by immunoprecipitation for 1 hr. using anti-FLAG antibody conjugated to Dynabeads (ThermoFisher Scientific). Proteins were eluted, dried and resuspended in SDS-loading buffer. Samples were alkylated with iodoacetamide and separated by SDS 10% PAGE. The entire lane was cut into sections and subjected to in-gel tryptic digestion. The isolated tryptic peptides were analyzed by LC-MS/MS using an ESI-LTQ XL mass spectrometer (ThermoScientific). Peptide identification was carried out using SEQUEST with a global false discovery rate of 5%.

### Flow cytometry

The experimental design and a detailed description of antibodies used for flow cytometry are provided in S1 Text and Table 2, respectively. Fibroblasts were infected with WT, *UL135*_*STOP*_, or *UL138*_*STOP*_ virus at 1 MOI. Alternatively, cells were lentivirally transduced with 1 MOI of _HA_Ub, *UL135*_*MYC*_, or *UL138*_*MYC*_. Cells were fixed with 2% paraformaldehyde in PBS for 30min and washed with excess PBS. For surface level experiments in fibroblasts, cells were stained with Brillant Violet 421-conjugated ms α-EGFR or APC-conjugated ms α-TNFR1 for 30min at 4°C in FACS buffer (PBS with 0.5% FBS). Samples were washed with excess FACS buffer to remove unbound antibody. Samples were washed with excess FACS buffer to remove unbound antibody.

**Table 2:** Antibody description and sources.

Antibody	Species	Source	Concentration
Alexa Fluor 546 anti rabbit	goat	Molecular Probes	IF: 1:7000
Alexa Fluor 647 anti mouse	goat	Molecular Probes	IF: 1:7000
Alexa Fluor 647 EGF ^a^	N/A	Molecular Probes	Flow 1μg / 1x10^5^ cells
APC conjugated TNFR1	mouse	R&D Systems	10 μL/10^6^ cells
Brilliant Violet 421 CD34	mouse	BioLegend	Flow 5 μL/ 1x10^6^ cells
Brilliant Violet 421 EGFR	mouse	BioLegend	Flow 5 μL/ 1x10^6^ cells
Dylight 700 conjugated anti mouse	goat	Pierce	Western: 1:12000
Dylight 800 conjugate anti rabbit	goat	Pierce	Western: 1:12000
EGFR (Ab-13)	mouse	Thermo Scientific	2μg per mg of lysate
EGFR (D38B1)	rabbit	Cell Signaling	Western 1:1000; IF 1:50
EGFR (MABF120)	mouse	Millipore	IF 1:50
Golgi GM130 (clone 35)	mouse	BD Transduction	IF 1:100
IE1/2 (3H4)	mouse	Tom Shenk; Princeton University	Western 1:1000
Myc epitope tag	chicken	GeneTex	Western 1:500
PDGFRα	rabbit	Cell Signaling	Western: 1:1000
PE conjugated CD34	mouse	BD Biosciences	Flow 20μL/1x10^6^ cells
phospho-EGFR (Tyr1068; D7A5)	rabbit	Cell Signaling	Western 1:1000; IF 1:50
Phosphotyrosine (4G10)	mouse	Millipore	Western 0.5μg/mL
pp28 (10B4-29)	mouse	Michael Nevels, University of Regensburg	IF 1:50
TGFβR1	Rabbit	Santa Cruz	Western 1:200
UL135	rabbit	Open Biosystems ^b^	Western 2 μg/mL
UL138	rabbit	Open Biosystems ^b^	Western 2 μg/mL
V5	mouse	Thermo Scientific	IP: 5 μg per 250 μg of lysate
V5	chicken	Bethyl	Western: 1:1000
VEGFR2	rabbit	Cell Signaling	Western: 1:1000
α-Tubulin (DM1A)	mouse	Sigma	Western 1:10000

^a^ Conjugated Ligand

^b^ Custom Ordered antibody

For trafficking experiments, fibroblasts were stimulated with 10nM EGF on ice for 30 minutes prior to staining for EGFR. Cells were washed with PBS and complete media warmed to 37°C was added to each sample. Samples were incubated at 37°C for 1-180min, washed with ice-cold PBS, and trypsinized on ice. Cells were processed as described above.

Detection of low level EGFR surface expression was optimized using HEK293T/17 cells (do not express endogenous EGFR) untransfected or transfected with pCIG-EGFR_3xFLAG_. 48h post transfection, cells were collected by trypsinization and stained using either Brilliant Violet 421 conjugated ms α-EGFR or Alexa Fluor 647 conjugate EGF ligand. Cells were analyzed using FACS to determine sensitivity and specificity (S2 Fig). For surface level experiments in CD34^+^ HPCs, infected (GFP+) HPCs were sorted using PE conjugated ms α-CD34 at 24 hpi (FACSAria, BD Bioscience Immunocytometry Systems). Cells maintained in LTBMC were labeled with Alexa Fluor 647-conjugated EGF at 1, 4, and 8dpi and fixed with 2% paraformaldehyde in PBS prior to FACS analysis. All samples were analyzed using a BD LSRII equipped with FACSDiva Software (BD Bioscience Immunocytometry Systems) and FlowJo software.

### Immunoprecipitation

Infected or lentivirus-transduced fibroblasts were lysed in TNEN lysis buffer (1% NP-40, 150 mM NaCl, 5 mM EDTA, 20 mM Tris-HCl pH 7.5, 10 mM sodium fluoride, 10 mM n-ethylmaleimide, 50 μM ammonia molybdate, 2 mM sodium orthovanadate with 1x HALT protease and phosphatase inhibitor cocktails (ThermoFisher)). EGFR was precipitated from 1mg of lysate, as determined using Pierce BCA assay kit (Thermo Fisher), using ms α-EGFR antibody (Ab13; Table 2) and Pierce Scientific protein G magnetic beads (Thermo Fisher). IP samples were washed with TNEN wash buffer (0.5% NP-40, 150 mM NaCl, 1 mM EDTA, 50 mM Tris-HCl pH 7.5, 10 mM sodium fluoride, 50 μM ammonia molybdate, 2 mM sodium orthovanadate) then resuspended in SDS-Page loading buffer with DTT for immunoblot analysis. Transfected HEK293T/17 cells were lysed using the above protocol. Immunoprecipitations were performed with 200 μg of protein lysate using ms α-V5 antibody. 20% of the lysates were analyzed to control for protein expression and should not be considered as a quantitative measure of relative protein levels.

For analysis of phosphorylation, infected cells were serum starved for 24h prior to lysis. At 48hpi, cells were incubated with complete media containing10nM EGF on ice for 30min and then prewarmed complete media at 37°C for 15min. Immunoprecipitations were performed as described above with 500μg of protein.

### Immunoblotting

Immunoprecipitation samples or 50μg of lysate were separated by electrophoresis on 10% Tris-Bis SDS-PAGE gel and either NuPage MES or MOPS buffers. Gels were transferred onto PVDF membrane (EMD Millipore) using NuPage Transfer buffer with 0.05% SDS and 20% methanol at 50V for 3h. Blots incubated with TBS-BT (Tris buffered saline with 0.25% BSA and 0.1% Tween20) for 30min prior to antibody staining. Rb α-EGFR(D38B1), chk α-Myc, chk α-V5, and ms α-phosphotyrosine (4G10) were incubated with the blots with 5% non-fat milk, 0.25% bovine serum albumin, and 0.05% sodium azide. In contrast, Ms α-IE1/2(3H4), rb α-phospho EGFR (pY1068), rb α-PDGFRα, and rb α-VEGFR2 were incubated with the blots in TBS with 0.1% Tween20, 5% BSA, and 0.01% Sodium azide, and ms α-Tubulin in TBS-BT. Blots were incubated with fluorescent secondary antibodies and imaged and quantitated using a Li-Cor Odyssey infrared system. Antibodies and sources are defined in Table 2.

### Immunofluorescence

For serum starvation condition, cells washed twice with PBS at 30hpi and incubated with serum-free media for 18h. Where specified, fibroblasts cells were transduced with 1 MOI of EGFR_3xFLAG_ lentivirus for 20h prior to CMV infection. Samples were processed as previously described and stained with antibodies (Table 2) [75]. All images were obtained using a DeltaVision RT inverted Deconvolution microscope. Representative single plane images were adjusted for brightness and contrast. Colocalization analysis was conducted using the Squassh workflow in the MosaicSuite for Image J and FiJi [31, 32].

### Measurement of infectious virus

Confluent fibroblasts were infected with 0.5 MOI of WT virus and were treated with DMSO, 5μM AG1478 (Caymen Chemical), or 20μM LY294002 (Cell Signaling) at 18 hpi. Virus present in cells and media were quantified by the TCID_50_ as described previously [10]. An infectious centers assay was used to quantitate infectious centers produced by CD34+ HPCs, as described previously [75]. CD34+ HPCs were treated with 10 μM AG1478, 10μM Gefitinib (Cell Signaling), 20 μM LY294002 or DMSO for vehicle control.

## Supporting Information

S1 Text Expanded experimental design and supplemental data results. (DOCX)

S1 Table EGFR and EGFR-associated proteins interacting with HCMV pUL138 during lytic infection. (TIF)

S1 Fig pUL138 increases TNFR cell surface levels. Fibroblasts were untransduced or transduced with lentivirus expressing either an empty vector or pUL138_MYC_. After 48h, cells were stained with APC conjugated ms α-TNFR1 to determine surface levels. Bar graphs represent the fold change compared to mock. Error bar represent SEM. Asterisks represents p-values<0.005 as calculated using the Student’s t-test.
(TIF)

S2 Fig Activated EGFR localizes to the viral assembly compartment during *UL135*_*STOP*_ and *UL138*_*STOP*_ infection. Fibroblasts infected with either UL135_STOP_ or UL138_STOP_ were stained with rb α-EGFR pY1068 and the golgi marker ms α-GM130. Cells were imaged by deconvolution microscopy. For all panels, nuclei are stained with DAPI. A merge of all three images in shown to the right.
(TIF)

S3 Fig Conjugated EGF ligand has increased sensitivity for detection of surface EGFR. (A) HEK293T/17 cells were transfected with EGFR_3xFLAG_. After 48h, cells were stained with either Brilliant Violet 421 ms α-EGFR (EGFR Ab) or Alexa Fluor 647 EGF ligand (EGF-647) and EGFR surface levels were analyzed by flow cytometry. (B) CD34^+^ cells were stained with EGF- 647 or EGFR Ab and EGFR surface levels were analyzed by flow cytometry. EGF-647 samples are an average of three experiments with SEM represented by error bars. EGFR-Ab for CD34^+^ is a representative experiment from three independent experiments using EGFR antibodies conjugated to a fluorescent tag. Bars represent the fold change relative to unstained controls.
(TIF)

S4 Fig Inhibition of EGFR with Gefitinib promotes reactivation from latency. Pure populations of CD34^+^ HPCs infected with WT, *UL135*_STOP_, or *UL138*_STOP_ at an MOI of 2 were isolated by FACS at 24 hpi and maintained in LTBMC with 10 μM of the EGFR inhibitor gefitinib. At 10 dpi, viable CD34^+^ HPCs were seeded by limiting dilution onto monolayers of permissive fibroblasts (reactivation). An equivalent number of cells were mechanically disrupted and seeded in parallel to determine the infectious virus present in the culture prior to reactivation (pre-reactivation). Frequency of infectious centers formed pre and post reactivation was determined 14 days later from the number of GFP-positive wells at each dilution using ELDA software. Bars represent the average fold change in gefitinib-treated cells relative to vehicle (DMSO) pre-reactivation control for three independent experiments. SEM is shown. Statistical significance was determined by two-way ANOVA with Bonferroni correction for differences between vehicle controls and drug treatments, asterisk indicates p-value<0.05.
(TIF)
