# Supplementary material for: Opposing Regulation of the EGF Receptor: A Molecular Switch Controlling Cytomegalovirus Latency and Replication
Source: PLoS Pathog. 2016 May 24;12(5):e1005655. doi: 10.1371/journal.ppat.1005655 (PMC4878804; doi:10.1371/journal.ppat.1005655)
Supplement: S1 Text — (DOCX) [file ppat.1005655.s001.docx]

**Supplemental Experimental Design**

**Flow Cytometry.** Detailed antibody information is included in Supplemental Tables 3. For flow cytometry with fibroblasts, MRC-5 cells infected with 1 MOI of WT, *UL135_STOP_,* or *UL138_STOP_* virus. Alternatively, cells were lentivirally transduced with 1 MOI of _HA_Ub, *UL135_MYC_*, *UL138_MYC_*, or empty vector. At 48hpi cells were washed with phosphate buffered saline (PBS), trypsinized, and pelleted in excess PBS. After an additional wash with PBS, cells were fixed with 2% paraformaldehyde in PBS for 30min and washed with excess PBS. Cells were stained with Brillant Violet 421 conjugated ms α-EGFR or APC conjugated ms α-TNFR1 for 30min at 4°C in FACS buffer (PBS with 0.5% FBS). Samples were washed with excess FACS buffer to remove unbound antibody.

For trafficking experiments, cells were washed with ice-cold PBS and incubated on ice for 30min. PBS was replaced with ice-cold serum free media containing 10nM EGF and cells were incubated on ice for 30min. Cells were washed with PBS and, except for the 0min time point samples, complete media at 37°C was added to each sample. Samples were incubated at 37°C for 1-180min, then washed with ice-cold PBS, and trypsinized on ice. Cells were processed as described above.

To analyze surface levels of EGFR on CD34^+^ HPCs, cells were infected with WT, *UL135*_STOP_, and *UL138*_STOP_. At 1dpi, cells were sorted by a FACSAria IIu (BD Bioscience Immunocytometry Systems) using Brilliant violet or PE conjugated ms α-CD34. At 1, 4, and 8 dpi 50,000-100,000 cells were then labeled with Alexa Fluor 647 conjugated EGF on ice for 30min. Cells were then washed with PBS and fixed with 2% paraformaldehyde in PBS before FACS analysis.

All samples were analyzed using a BD LSRII equipped with FACSDiva Software (BD Bioscience Immunocytometry Systems). Post-cytometry analysis was performed with FlowJo software (FlowJo) and Microsoft Excel (Microsoft).

**Optimization of EGFR detection:** HEK293T/17 cells were transfected with pCIG-EGFR_3xFLAG_. 48h post transfection, cells were collected by trypsinization and stained using either Brilliant Violet 421 conjugated ms α-EGFR or Alexa Fluor 647 conjugate EGF ligand. Cells were analyzed using FACS. Staining protocol for CD34^+^ cells is in the material and methods section of the paper.

**Supplemental Results**

**UL138 alone upregulates TNFR surface levels.**  Upregulation of TNFR surface levels by pUL138 has been previously described by a couple of different lab ([Le et al., 2011](#_ENREF_1); [Montag et al., 2011](#_ENREF_2)). EGFR surface levels are decreased during infection of fibroblasts in the absence of *UL138* (Fig. 2B). However, unlike in the case of TNFR1, pUL138 expression alone did not upregulate surface levels of EGFR (Fig. 2C). Therefore, we wanted to confirm that pUL138 alone upregulated TNFR surface levels using our experimental conditions. We stained fibroblasts lentivirally transduced to overexpress pUL138myc for surface TNFR1 with specific APC conjugated antibody (Fig. S1). As previously described, expression of UL138 increased TNFR when compared to either mock-treated or empty vector transduced samples (p-value<0.005). Because pUL138 alone increases TNFR1, but not EGFR, distinct mechanism may be involved or at the very least other viral or infection-induced factors are required for the regulation of EGFR.

**Detection of EGFR with conjugated EGF ligand is more sensitive.** CD34^+^ HPCs express low levels of EGFR on their cell surface. To enhance detection of EGFR and discern infection-related changes in surface expression of EGFR in CD34+ HPCs, we used EGF ligand conjugated to an Alexa Fluor-647 fluorescent tag (EGF-647) to heighten sensitivity. To verify the specificity and sensitivity of this assay, we used human embryonic kidney-293 cells, which do not express EGFR ([Uchida et al., 2007](#_ENREF_3)). HEK-293 cells were transfected with a plasmid expressing EGFR with a 3xFLAG. The cells were then stained using either a Brilliant Violet 421 mouse α-EGFR antibody or an EGF ligand. The reason for using the EGF ligand is that, to our knowledge, EGF only binds to EGFR and has not been reported to bind to other ErbB surface receptors. Using the conjugated ligand, we detected a ~2500-fold increase over unstained samples, whereas the fluorescently conjugated antibody for EGFR increased 800-fold (Fig. S2). Conjugated EGF staining of untransfected controls indicated no loss of specificity. These results suggest that the conjugated ligand provides enhanced sensitivity for detecting low surface levels of EGFR without a loss of specificity.

To determine if the optimized protocol would work on CD34^+^ cells, we stained pools of CD34^+^ cells in with EGF-647 and analyzed them by FACS ( Fig. S2B). Using the ligand we can detect a 5-fold increase of the geometric mean of fluorescence intensity compared to unstained sample. In contrast, no difference was detected when using BV-421- or PE-conjugated ms α-EGFR antibodies (Fig. S2B and data not shown). This demonstrates the superiority of using EGF-conjugated ligand for detection of EGFR on CD34^+^ cell in comparison to traditional conjugated EGFR antibodies.

**References**

Le, V.T., Trilling, M., and Hengel, H. (2011). The Cytomegaloviral Protein pUL138 Acts as Potentiator of TNF Receptor 1 Surface Density to Enhance ULb'-encoded modulation of TNF-{alpha} Signaling. J Virol.

Montag, C., Wagner, J.A., Gruska, I., Vetter, B., Wiebusch, L., and Hagemeier, C. (2011). The latency-associated UL138 gene product of human cytomegalovirus sensitizes cells to tumor necrosis factor alpha (TNF-alpha) signaling by upregulating TNF-alpha receptor 1 cell surface expression. J Virol *85*, 11409-11421.

Uchida, A., Hirano, S., Kitao, H., Ogino, A., Rai, K., Toyooka, S., Takigawa, N., Tabata, M., Takata, M., Kiura, K.*, et al.* (2007). Activation of downstream epidermal growth factor receptor (EGFR) signaling provides gefitinib-resistance in cells carrying EGFR mutation. Cancer science *98*, 357-363.
