# Supplementary figures and images for: Opposing Regulation of the EGF Receptor: A Molecular Switch Controlling Cytomegalovirus Latency and Replication
Source: PLoS Pathog. 2016 May 24;12(5):e1005655. doi: 10.1371/journal.ppat.1005655 (PMC4878804; doi:10.1371/journal.ppat.1005655)

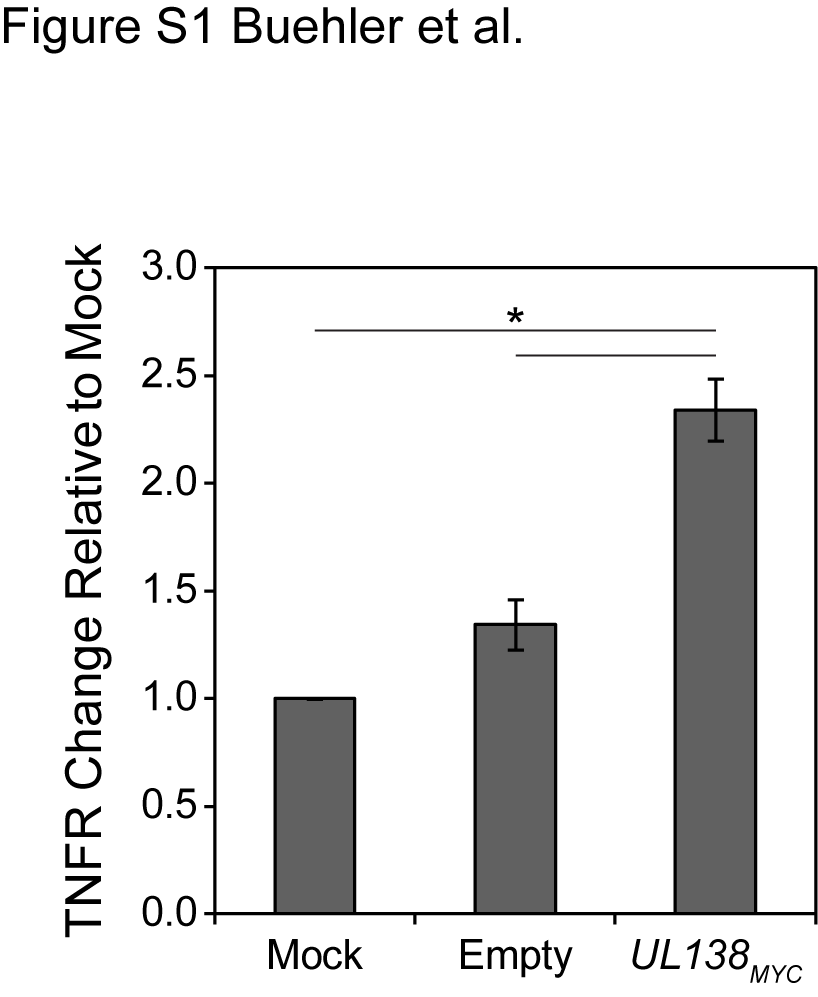

Supplement: S1 Fig — Fibroblasts were untransduced or transduced with lentivirus expressing either an empty vector or pUL138MYC. After 48h, cells were stained with APC conjugated ms α-TNFR1 to determine surface levels. Bar graphs represent the fold change compared to mock. Error bar represent SEM. Asterisks represents p-values<0.005 as calculated using the Student’s t-test. (TIF) [file ppat.1005655.s003.tif]

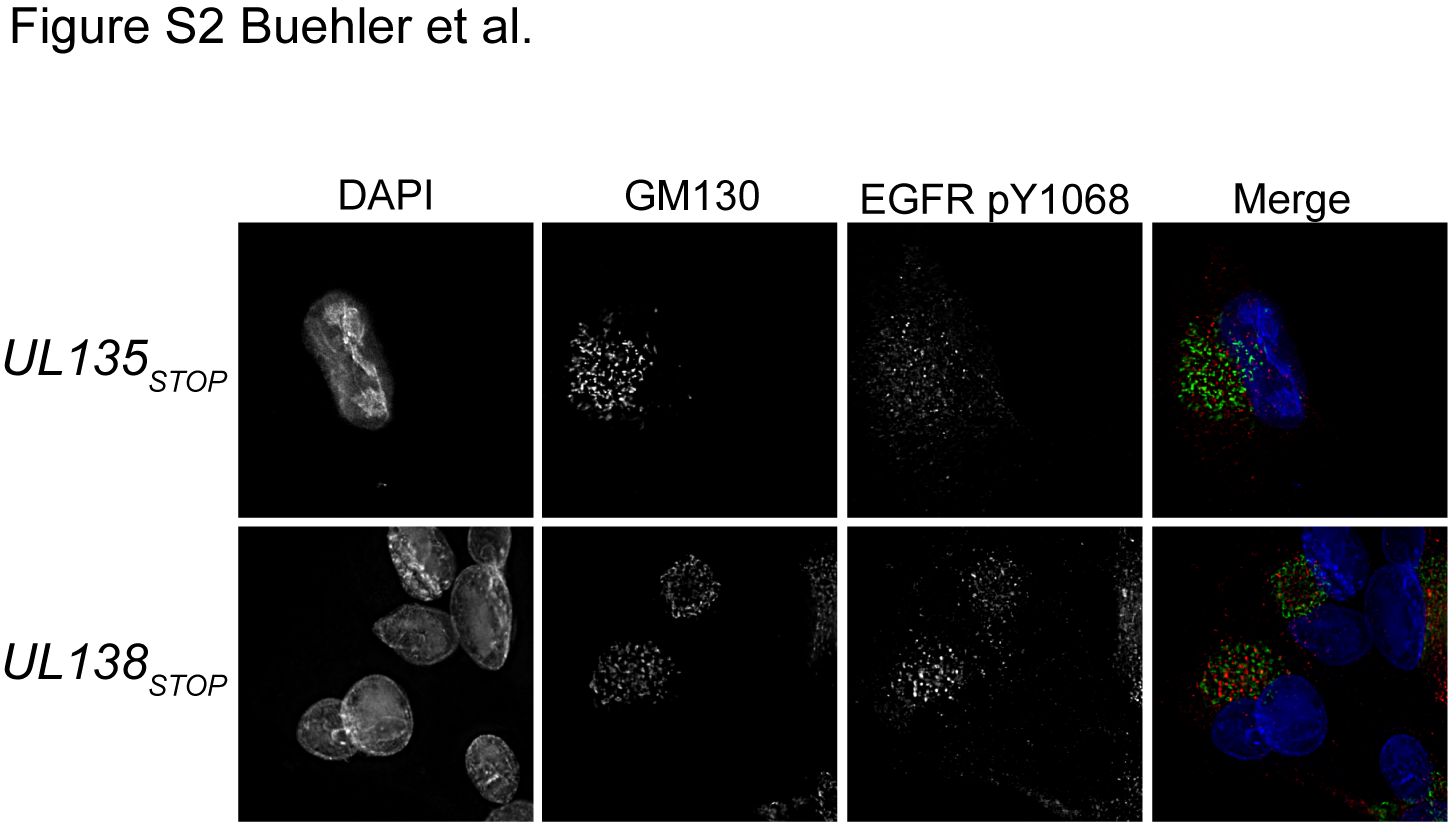

Supplement: S2 Fig — Fibroblasts infected with either UL135STOP or UL138STOP were stained with rb α-EGFR pY1068 and the golgi marker ms α-GM130. Cells were imaged by deconvolution microscopy. For all panels, nuclei are stained with DAPI. A merge of all three images in shown to the right. (TIF) [file ppat.1005655.s004.tif]

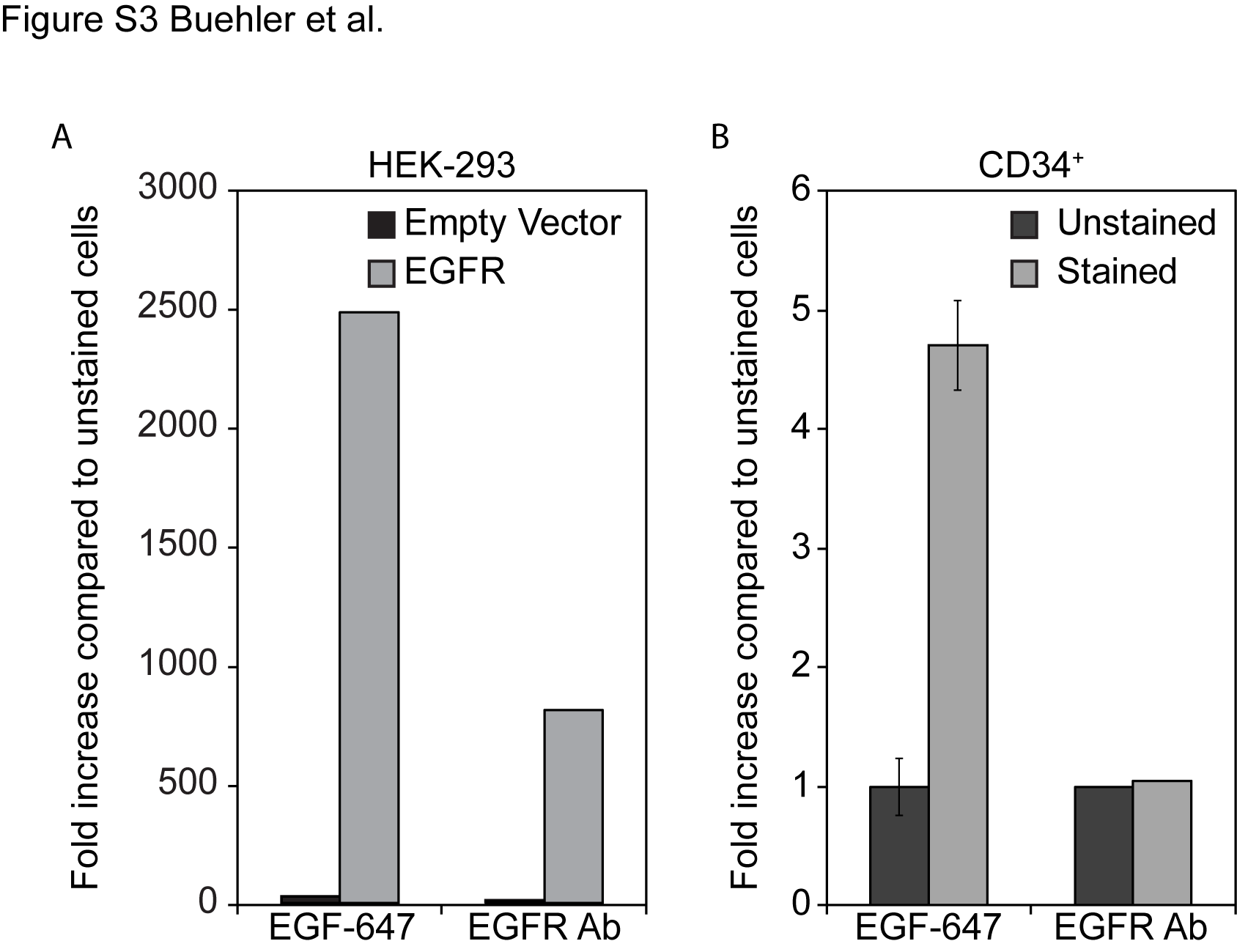

Supplement: S3 Fig — (A) HEK293T/17 cells were transfected with EGFR3xFLAG. After 48h, cells were stained with either Brilliant Violet 421 ms α-EGFR (EGFR Ab) or Alexa Fluor 647 EGF ligand (EGF-647) and EGFR surface levels were analyzed by flow cytometry. (B) CD34+ cells were stained with EGF- 647 or EGFR Ab and EGFR surface levels were analyzed by flow cytometry. EGF-647 samples are an average of three experiments with SEM represented by error bars. EGFR-Ab for CD34+ is a representative experiment from three independent experiments using EGFR antibodies conjugated to a fluorescent tag. Bars represent the fold change relative to unstained controls. (TIF) [file ppat.1005655.s005.tif]

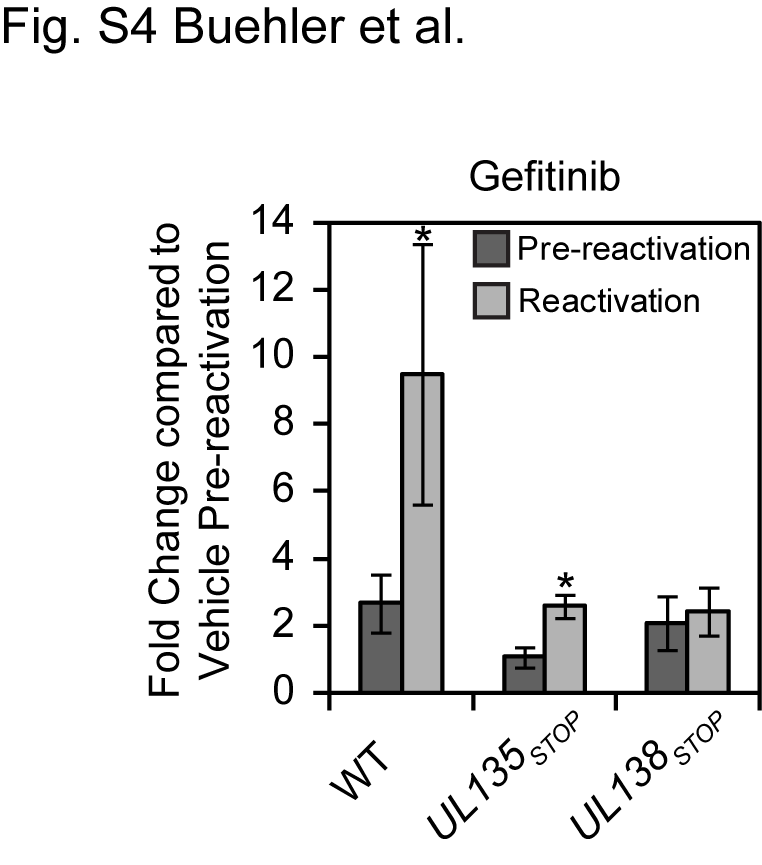

Supplement: S4 Fig — Pure populations of CD34+ HPCs infected with WT, UL135 STOP, or UL138 STOP at an MOI of 2 were isolated by FACS at 24 hpi and maintained in LTBMC with 10 μM of the EGFR inhibitor gefitinib. At 10 dpi, viable CD34+ HPCs were seeded by limiting dilution onto monolayers of permissive fibroblasts (reactivation). An equivalent number of cells were mechanically disrupted and seeded in parallel to determine the infectious virus present in the culture prior to reactivation (pre-reactivation). Frequency of infectious centers formed pre and post reactivation was determined 14 days later from the number of GFP-positive wells at each dilution using ELDA software. Bars represent the average fold change in gefitinib-treated cells relative to vehicle (DMSO) pre-reactivation control for three independent experiments. SEM is shown. Statistical significance was determined by two-way ANOVA with Bonferroni correction for differences between vehicle controls and drug treatments, asterisk indicates p-value<0.05. (TIF) [file ppat.1005655.s006.tif]
